# Development of Ensemble Steric and Electrostatic Chirality (ESEC) descriptors for modelling chromatographic enantioseparations

**DOI:** 10.1371/journal.pone.0333635

**Published:** 2025-10-17

**Authors:** Jordy Peeters, Pieter De Gauquier, Fardine Ameli, Yvan Vander Heyden, Debby Mangelings, Kenno Vanommeslaeghe

**Affiliations:** Faculty of Medicine and Pharmacy, Department of Analytical Chemistry, Applied Chemometrics and Molecular Modelling, Vrije Universiteit Brussel (VUB), Brussels, Belgium; Inonu University, Faculty of Pharmacy, TÜRKIYE

## Abstract

In this work, chiral molecular descriptors were defined using 2 distinct approaches: (1) scalar triple products of vectorial molecular properties, and (2) descriptors that attempt to quantify the amount of twist in the overall molecular shape. Because both approaches give rise to conformation dependence, descriptor values were averaged over a conformational ensemble obtained by Molecular Dynamics. In addition, a method is introduced that attempts to quantify the asymmetry of the distribution of the descriptor values over the conformational ensemble. The totality of the resulting descriptors were named “Ensemble Steric and Electrostatic Chirality (ESEC) descriptors”. A pilot validation study was performed by building Quantitative Structure-Enantioselectivity Relationships (QSER), i.e. mathematical models to predict the chromatographic separation of enantiomers, using a test set of 43 structurally diverse pharmaceuticals analyzed on a polysaccharide-based chiral stationary phase. The best linear regression model (7 descriptors) for the chiral separation (expressed as selectivity factor) featured a low leave-one-out cross validation error (0.0814), a well-predicted elution sequence of the separated enantiomers (21 out of 23 molecules) and a well-predicted *α*_*RS*_ for 27 out of 42 molecules. To the best of our knowledge, this is the first time that acceptable linear QSER models were obtained for chiral chromatographic separations of such a chemically diverse set of pharmaceuticals.

## 1. Introduction

A molecular descriptor is defined as either the result of some standardized experiment or as a useful number obtained from a mathematical procedure that operates on a molecule’s chemical representation [1]. They can be used to predict physical, chemical or biological properties of molecules, usually by means of a regression model [2]. In the specific case of chiral properties, descriptors that distinguish between enantiomers, i.e. chiral descriptors, need to be used. For predicting retention on a chiral chromatographic system, the regression models are called Quantitative Structure-Enantioselective Retention Relationships (QSERR) [3,4]. In this context, building proper models is challenging, because the interactions between the chiral stationary phase (CSP) and the enantiomers are complex [2]. Therefore, the *chiral* descriptors used for this purpose do not only need to capture the physics of the analyte-stationary phase interactions, but more specifically the 3D *asymmetry of these interactions*. Despite substantial efforts of several research groups to develop suitable chiral descriptors [4], the latter remain relatively scarce, hard-to-interpret, and only yield satisfactory results when applied to highly congeneric series of analytes [5–7]. For an overview of chiral descriptors applied to chiral separations on derivatized polysaccharide and macrocyclic antibiotic CSP, we refer to [2]. Additionally, various approaches were employed to predict enantioselectivity, such as atomistic calculations (docking and Molecular Dynamics (MD)) and empirical fitting. For more information, we also refer to [2].

Because of stereochemical differences between the enantiomers, each enantiomer may interact differently with the chiral selector of a CSP leading to different retentions and separation. Chiral descriptors take these differences into account and therefore should be able to distinguish between enantiomers. On polysaccharide-based CSP, multiple non-covalent interactions play a role, including electrostatic and hydrophobic interactions, in general, and hydrogen bonds, π-π interactions, and halogen bonds in particular [2,8,9]. During chromatography, enantiomers continuously exchange between the CSP and mobile phase, forming transient diastereomeric complexes [10]. For each enantiomer of a pair, the interaction energy (*∆G*) with the CSP may be different, leading to the formation of a more stable complex with one enantiomer. These *∆G* values can be related to the separation factor (*α*) by Eq 1 [10]:


ΔG = −RT ln α (1)

Accordingly, we will build Quantitative Structure-Enantioselectivity Relationship (QSER) models for ln *α*, where −*∆G*/R*T* is a linear combination of chiral descriptors.

Unlike common molecular properties that are often used in (achiral) Quantitative Structure-Retention Relationship (QSRR) modelling, chirality is by definition 3D. In the most general sense, chiral properties are even *inherently conformation-dependent*. The most prominent manifestation of this conformation-dependence is atropisomerism, where two distinct enantiomers are merely separated by a *conformational* barrier to rotation [11]. Specifically, LaPlante has classified atropisomers by their interconversion half-life at 37 °C in class 1 (*t*_1/2_ < 60 s), class 2 (60 s < *t*_1/2_ < 4.5 years), and class 3 (*t*_1/2_ > 4.5 years) [12,13]. Examples include 6,6’-dini*t*ro-2,2’-diphenic acid and BINOL (S1 Fig (Supporting Informa*t*ion)) [14–16]. Furthermore, even molecules that are asymmetric because of a chiral center often exhibit fundamental chiral properties (such as Circular Dichroism spectra) that are highly solvent and sometimes even temperature-dependent, clearly demonstrating that chiral properties cannot be determined from 2D information alone. Although conformation-dependent chiral descriptors do exist [17], they are often difficult to interpret or not generally applicable.

In a previous pilot study [18], models were built for 18 structurally diverse chiral molecules, analyzed on different chromatographic systems, to predict the retention, separation and elution sequence of enantiomers. Five new chiral descriptors, consisting of vectors that represent fundamental molecular properties, were used to construct these chiral models. However, this initial study did not yield satisfactory chiral models. One of the main limitations of these first-generation chiral descriptors was their lack of robustness, attributed to the use of higher-order functions.

Accordingly, the goal of the present study is to propose novel and better chiral descriptors based on tangible physical properties of the analyte. QSER models are then built from these descriptors to demonstrate their viability.

The first class of conformation-dependent chiral descriptors is based on the scalar triple product of three spatial vectors derived from an enantiomer’s atomic constellation. This product’s magnitude equals the volume of the parallelepiped formed by these vectors, with opposite signs for two enantiomers. For symmetrical molecules, the vectors lie in the symmetry plane, making the triple product zero. This approach aligns with the work of Dervarics et al. [19], who used displacement vectors between pharmacophores in QSAR (Quantitative Structure-Activity Relationship) models. Since these vectors are related to noncovalent interactions, they are easy to understand and provide insights into enantiorecognition. However, Dervarics’ approach requires the same pharmacophores across all molecules, limiting applicability to congeneric series of compounds. Viewed in this context, our first family of descriptors is similar but made more universal by using spatial vectors independent of specific chemical groups.

While the chiral recognition mechanism between a polysaccharide-based CSP and a chiral molecule is not fully understood, it is believed that the CSP adopts a helical structure featuring hydrophobic grooves favoring one enantiomer [20,21]. Accordingly, a second class of conformation-dependent chiral descriptors was constructed, which attempts to quantify to which extent the shape of the molecule exhibits a (pseudo)helical twist. Like the “triple product descriptors”, these “shape-based descriptors” have opposite signs for mirror-image conformations; see §2.1 for details.

Since drug-like molecules generally possess conformational flexibility, the conformation-dependent descriptors are calculated on a conformational ensemble from an MD simulation in an environment mimicking the mobile phase (see §3.1.1), capturing analyte and solvent effects. As is customary in QSER, information about the *stationary phase* is excluded, increasing workflow flexibility because the precise microscopic structures of CSPs are often unclear. The final conformation-*independent* descriptor is obtained by averaging the analyte’s conformation-dependent descriptors across its conformational ensemble, called the “averaged” descriptors. Additionally, “windowed” descriptors are computed by applying a pair of window functions to the conformation-dependent descriptor distribution, yielding a pair of descriptors reflecting the compound’s preferential conformations (see §2.2).

**Fig 1:**
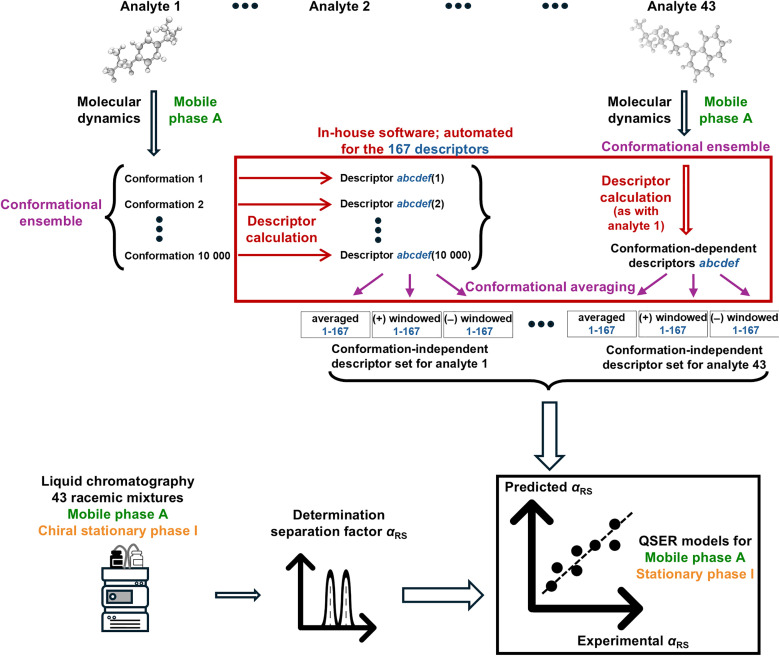
Scheme for constructing QSERR models based on the separation factors, *α*_RS_, for 43 racemates.

Combining the ‘triple product’ and ‘twist’ descriptors yields 167 conformation-*dependent* chiral descriptors (see Fig 1). When applied to a conformational ensemble to generate conformation-*independent* descriptors, 167 corresponding “averaged” and twice as many (334) “windowed” descriptors are generated, bringing the total number of our newly defined Ensemble Steric and Electrostatic Chirality (ESEC) descriptors to 501. Taking into account different conformational ensembles based on different solvent models (as elaborated in §3.3), eight sets of 501 descriptor values were obtained and subsequently validated for building QSER models.

In summary, the conformational ensemble of each of the 43 enantiomers was determined by means of MD simulations. Using in-house software, the conformation-dependent descriptor values were calculated on each MD snapshot. These values are then averaged over the molecule’s conformational ensemble via two different methods resulting in the averaged and windowed “conformation-independent” descriptors; see theory §2.2. These (sub)sets of predictor variables are then applied to build QSER models by sMLR.

The experimental chromatographic data used in these models consisted of selectivity factors for 43 structurally diverse commercially available chiral drugs (Fig 1). Each racemic analyte was run on a Lux amylose-2 CSP (amylose tris(5-chloro-2-methylphenylcarbamate) selector) in reversed-phase liquid chromatography (RPLC) mode using a basic mobile phase. In addition, the elution sequence was determined by analyzing the individual enantiomers. Since the present paper mainly focuses on the definition and calculation of the descriptors, models were built for only one chromatographic system (= one stationary phase with one mobile phase) as a proof-of-concept. A manuscript is in preparation applying the same descriptors to multiple chromatographic systems.

## 2. Theory

### 2.1. Construction of conformation-dependent chiral descriptors

a **Triple products of vectors denoting distributions of atomic properties**

As briefly mentioned in the introduction, chiral descriptors in this class are based on scalar triple products of three different first-order moment vectors. A well-known example of such a vector is the molecular dipole moment (Eq 2):


$$\mu =\;\sum_{\text{atoms }i}q_{i}\;{𝐫}_{𝐢}$$ (2)

With $q_{i}$ the atomic partial charge and ${𝐫}_{𝐢}$ the position vector of atom i. Similar vectors were constructed by replacing the partial charge by another atomic attribute, $x_{i}$, which may, for example, be a measure for the atom’s polarizability, its ability to form hydrogen bonds,… (Eq 3).


$${𝐯}_{𝐱}=\;\sum_{\text{atoms }i}x_{i}\;{𝐫}_{𝐢}$$ (3)

It is important to note that, when $\sum_{i}x_{i}\neq \text{0}$, the resulting moment ${𝐯}_{𝐱}$ becomes translationally variant; this is a well-known problem when attempting to calculate the dipole moment of a molecule with a nonzero net charge [22]. In this work, we mitigate this issue by translating the center of mass of any given molecular geometry to the origin prior to calculating its moment vectors. The center of mass is one of the more physically meaningful options in this respect, because it is the only choice that yields a dipole moment that is proportional to the torque an unrestrained molecule will undergo in an electric field [23].

An overview of the proposed vectors is given in Table 1. However, vector ${𝐯}_{𝐦𝐬}$ is not calculated according to Eq 3. Indeed, ${𝐯}_{𝐦𝐬}$, as obtained by setting $x_{i}=m_{i}$, is simply the position vector of the center of mass, which in our case will always be zero due to the method chosen to mitigate the translational variance of all other moments. Instead, we use a measure for the asymmetry of the mass distribution ${𝐯}_{𝐦𝐬}$ as defined in Eq 4:

**Table 1:** Overview of atomic attribute-based vectors used in computing the scalar triple product to obtain the chiral descriptor.

Name vector	Attribute (*X*_*i*_)	Description
${𝐯}_{𝐦𝐬}$	Mass*	Atomic mass
${𝐯}_{𝐜𝐡}$	Charge	CGenFF partial charge
${𝐯}_{𝐚𝐜}$	Absolute charge	Absolute value of atomic partial charge
${𝐯}_{𝐠𝐬}$	Gasteiger charge	Partial charge computed via Gasteiger algorithm
${𝐯}_{𝐚𝐠}$	Absolute Gasteiger charge	Absolute atomic partial charge obtained from Gasteiger algorithm
${𝐯}_{𝐚𝐥}$	Polarizability	Atomic polarizability (“alpha”)
${𝐯}_{𝐩𝐢}$	p-orbital	Value of 1 is assigned to atoms having a p-orbital
${𝐯}_{𝐡𝐝}$	Hydrogen bond donor (HBD)	Value of 1 is assigned to H-atoms which can form a HB
${𝐯}_{𝐡𝐚}$	Hydrogen bond acceptor (HBA)	Value of 1 is assigned to HB-accepting atoms
${𝐯}_{𝐡𝐛}$	HBD + HBA	Value of 1 is assigned to all atoms which can form HBs
${𝐯}_{𝐝𝐚}$	HBD – HBA	Value of 1 is assigned to HB-donating atoms and −1 to HB-acceptors

* ${𝐯}_{𝐦𝐬}$ is calculated differently from the other vectors; see text.


$${𝐯}_{𝐦𝐬}=\frac{\;\sum_{\text{atoms }i}m_{i}\;{𝐫}_{𝐢}\left(m_{i}\left\|{𝐫}_{\text{i}}\right\|\right)}{\sum_{\text{atoms }i}\left(m_{i}\left\|{𝐫}_{\text{i}}\right\|\right)}$$ (4)

This is inspired by our pilot study [18], where Eq 4 was used more systematically but the robustness of the resulting descriptors with respect to small conformational changes was observed to be poor. Therefore, in the present study, we limit ourselves to the first-order moments defined in Eq 3, with the exception for ${𝐯}_{𝐦𝐬}$.

As mentioned above, when partial charges are used for $x_{i}$, the molecular dipole moment is obtained. By taking the absolute values of these charges instead, a vector corresponding to the distribution of polar groups is obtained. Since atomic partial charges are not rigidly defined, we chose to compute these vectors using either the partial charges from version 1.1.0 of the Charmm General Force Field (CGenFF) program [24] or the Gasteiger-Marsili charges obtained from OpenBabel version 3.1.1 [25]. This resulted in two different values for **µ**, the molecular dipole moment (${𝐯}_{𝐜𝐡}$ and ${𝐯}_{𝐠𝐬}$) and for the vector for polarity (${𝐯}_{𝐚𝐜}$ and ${𝐯}_{𝐚𝐠}$). To include a proxy for the strength of the London dispersion interactions, the fitted atomic polarizabilities from Wang et al. (AA-models) [26] were used for calculating ${𝐯}_{𝐚𝐥}$. Since π interactions between the analyte and the CSP are important, a vector ${𝐯}_{𝐩𝐢}$ was introduced. The attribute *x*_*i,pi*_ used for this purpose was set to 1 for atoms with an aromatic CGenFF 1.1.0 atom type [27] and 0 for all other atoms. Similarly, four vectors were defined based on the hydrogen bond donating and accepting character of atoms as determined by their CGenFF atom type. In the case of ${𝐯}_{𝐡𝐝}$, hydrogen atoms that are capable of forming hydrogen bonds (HB) get a value for *x*_*i,hd*_ equal to 1. Similarly, the *x*_*i,ha*_ of HB-accepting atoms was set to 1 for the purpose of constructing ${𝐯}_{𝐡𝐚}$. The attribute *x*_*i,hb*_ was set to 1 for both HB-donating and -accepting atoms ${(𝐯}_{𝐡𝐛}={𝐯}_{𝐡𝐚}+{𝐯}_{𝐡𝐝})$. Finally, ${𝐯}_{𝐝𝐚}$ was constructed the same way but with a value of −1 for HB-accepting atoms instead ${(𝐯}_{𝐝𝐚}={𝐯}_{𝐡𝐚}-\;{𝐯}_{𝐡𝐝})$.

Based on the 11 vectors defined in Table 1, 11!/ (8! 3!) = 165 unique triple products could be constructed, each of which would constitute a chiral descriptor. To keep track of the physical meaning of all these descriptors, the 2-letter abbreviations of Table 1 were applied to indicate which vectors they consist of. For instance, descriptor *msagpi* is obtained by taking the scalar triple product of ${𝐯}_{𝐦𝐬}$, ${𝐯}_{𝐚𝐠}$, and ${𝐯}_{𝐩𝐢}$. More generally, an arbitrary descriptor named *abcdef* is defined as


$$\begin{array}{c} abcdef\mathrm{\backslash stackrel}def=\frac{\left({𝐯}_{𝐚𝐛}\times {𝐯}_{𝐜𝐝}\right)\cdot {𝐯}_{𝐞𝐟}}{{\left(\left\|{𝐯}_{𝐚𝐛}\right\|\left\|{𝐯}_{𝐜𝐝}\right\|\left\|{𝐯}_{𝐞𝐟}\right\|\right)}^{\frac{\text{2}}{\text{3}}}} \end{array}$$ (5)

where the denominator is inspired by Dervarics et al. [19] and serves to reduce the dimension of the final descriptor value to a length, with which we hope to further mitigate the lack of robustness that was encountered with the more naive triple product descriptors in our pilot study [18]. Finally, it should be noted that some combinations of vectors are not expected to yield sensible results, for example those including two copies of the same vector based on different charge models (CGenFF vs. Gasteiger). Excluding such combinations, a total of 143 pure triple products of distributions of atomic properties were available for model building in the present paper. To this, 24 descriptors were added that contain shape-based properties, as outlined in the next subsection.

b **Shape-based descriptors**

A second class of descriptors, introduced in the present study, is loosely based upon a combination of (1) the chiral recognition model proposed by Booth et al. in 1997 [20], and (2) the notion that polysaccharide-based chiral selectors present the analytes with a helical binding groove, as first proposed by Yamamoto et al. in 2002 [21]. The picture emerging from these references is that the chiral recognition process is not necessarily driven by the analyte, exhibiting different non-bonded properties when viewed from different angles, but may (also) be the result of general shape complementarity of the analyte with the aforementioned helical binding groove (or “ravine” in Booth’s terminology). Accordingly, we sought to develop descriptors that capture the degree to which the general shape of an arbitrary organic molecule exhibits a “helix-like” twist. As measures of helicity are intrinsically linked to dihedral angles, the most straightforward measure of twist would be a dihedral angle of the whole shape of the molecule. However, mathematically spoken, the calculation of a dihedral angle in a molecular context requires four points in space, necessitating a representation of the analyte as a chain of four “blobs” (which conceptually bear some degree of analogy to particles in Coarse Grained force fields [28]). This was accomplished by clustering the atomic coordinates of an arbitrary molecular conformation into four clusters, upon which our shape-based descriptors are built.

**Fig 2:**
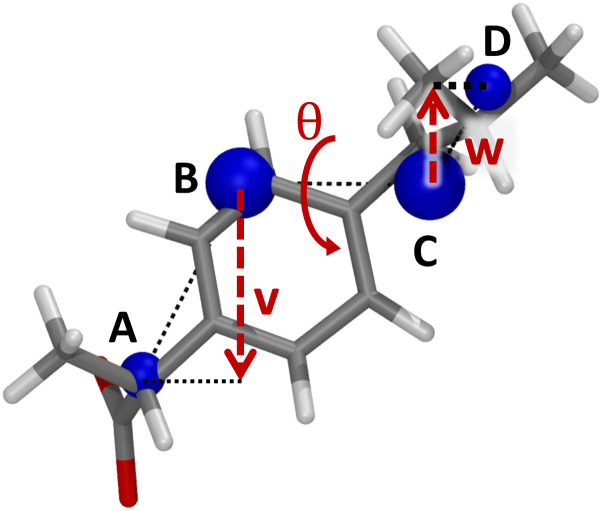
A conformation of ibuprofen obtained from the MD simulation with its four cluster centers A-D (blue).

Firstly, a complete linkage spatial clustering [29] of all atomic coordinates is performed, setting the clustering cut-off such that exactly four clusters are obtained. Next, the geometric means of all atomic coordinates in each cluster are computed. As an example, the cluster centers for (an arbitrary conformation of) ibuprofen are displayed in Fig 2. Subsequently, the centers which are the furthest away from one another are labelled A and D. Finally, the two remaining centers are labelled B and C such that $\frac{\left|AB\right|}{\left|AC\right|}<\frac{\left|BD\right|}{\left|CD\right|}$. This ordering of the cluster centers is generally rigorous, except that the choice between ABCD and the inverse (DCBA) is arbitrary; how this is handled is discussed below.

A descriptor was defined that aims at representing the three-dimensional twist of the compound more explicitly. As hinted above, this descriptor is based on the dihedral angle of cluster centers A, B, C and D (Fig 2). For two mirror-image geometries, this dihedral angle will have the same absolute value but opposite sign. In addition, the closer it approaches 90°, the greater the out-of-plane angle. Combining these two properties, the sine of this dihedral angle seems a natural factor to be incorporated into a chiral descriptor, representing the molecular twist. Note that inverting the sequence (DCBA) will not affect this sign. In order for our final descriptor *stwist* to have the dimension of a length (which should be advantageous for robustness as argued above), we multiplied the sine of the dihedral angle ABCD, θ_ABCD_, with (both) the distances of A and D to the BC axis (denoted as **v** and **w** respectively) and then divided by the magnitude of the vector **q** from point B to point C (**q** = $\vec{BC}$). This mathematical process is described by Equation 6, with the relevant quantities illustrated in Fig 2 displayed on an arbitrary conformation of ibuprofen.


$$stwist=\frac{\left\|𝐯\right\|\cdot \left\|𝐰\right\|\text{sin}\;\theta }{\left\|𝐪\right\|}$$ (6)

Finally, Booth et al. [20] specifically assume a combination of a hydrophilic anchor point with *hydrophobic interactions between the analyte and the binding ravine/groove*. This assumption is confirmed by the work of Yamamoto et al. [21] and Wang et al. [30]. Accordingly, a variant of the “twist” descriptor was calculated based on a clustering of only the hydrophobic atomic coordinates, rather than all atoms (as inferred from their CGenFF atom types). This resulted in the descriptor *ftwist.*

c **Triple product-based descriptors combining molecular shape and atomic properties**

A last manner we propose to develop chiral descriptors is by combing the strategy from the triple product with the shape based one. By exploiting the clustering of the atomic coordinates, we proposed an additional pair of vectors which describes the molecule’s shape. Based on the ordering described in the previous paragraph, an outer and inner shape vector could be defined as ${𝐯}_{𝐬𝐨}=\vec{AD}$ and ${𝐯}_{𝐬𝐢}=\vec{BC}$, respectively. These two shape vectors can then be combined with a third vector from Table 1, giving rise to 11 additional descriptors of the form *sosixx* (i.e., scalar triple products of ${𝐯}_{𝐬𝐨}$, ${𝐯}_{𝐬𝐢}$ and a vector ${𝐯}_{𝐱𝐱}$ from Table 1). Note how inverting the sequence ABCD inverts the signs of *both* ${𝐯}_{𝐬𝐨}$ and ${𝐯}_{𝐬𝐢}$, so that the final value of the triple product is unchanged. An additional pair of vectors was defined but based on the hydrophobic shape of the molecule using the same methodology as in which only the hydrophobic atoms were clustered. More specifically, 11 descriptors of the form *xxfifo* were constructed using vectors ${𝐯}_{𝐟𝐨}$ and ${𝐯}_{𝐟𝐢}$, but using the cluster centers of only the hydrophobic atoms.

### 2.2. Development of averaged and windowed conformation-independent chiral descriptors

**Fig 3:**
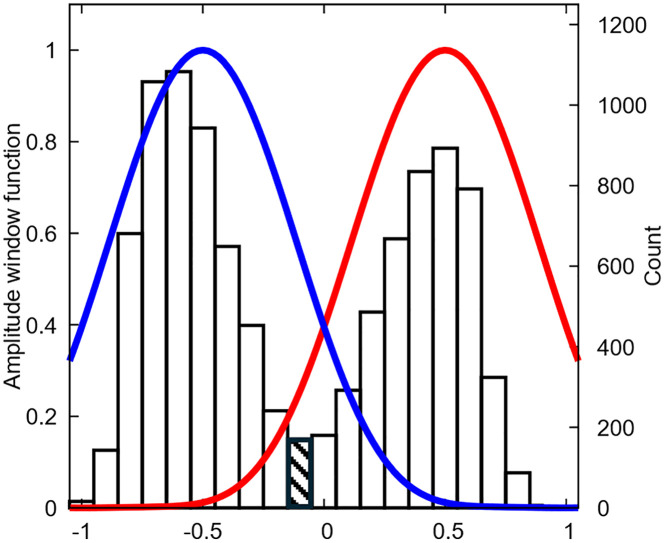
Horizontally scaled histogram 𝐇(𝐱) of the descriptor stwist of (R)-ibuprofen (obtained from the explicit solvent simulation. See §2.1b). The averaged descriptor value, i.e., the average value, is situated in the shaded box. The (+) and (-) windowed descriptor values are obtained when the product between H(x) and the positive (red) or negative (blue) windowed window functions, f+(x) or f−(x), respectively, is integrated.

As mentioned in the introduction, two variants of conformation-independent descriptors are proposed. The “averaged” descriptors are simple ensemble averages of the corresponding conformation-dependent descriptors. Conversely, to calculate a “windowed” version of an arbitrary descriptor *abcdef*, the descriptor values *abcdef*(*t*) of the individual snapshots *t* are first scaled by a factor *s* such that max|*s*.*abcdef*(*t*)| = 1. Consequently, the (normalized) histogram H(*x*) of the scaled descriptor *x* = *s*.*abcdef*(*t*)) is horizontally bound between −1 and 1, as can be seen on the example in Fig 3.

To this histogram, either the positive window function $f^{+}(x)$ or the negative window function $f^{-}(x)$ may be applied via Eq 7, giving rise to the final positive- and negative-windowed descriptors *abcdef*+ and *abcdef* − , respectively.


$$abcdef\pm \;=\int_{-\text{1}}^{\text{1}}f^{\pm }(x)H(x)dx=\;\frac{\text{1}}{n}\sum_{t=\text{1}}^{n}f^{\pm }\left(s.abcdef(t)\right)$$ (7)

As for the mathematical description of the window function and its derivation, we refer to the Supporting Information (S1 File). Note that for a converged simulation of an *achiral* molecule, *abcdef*(*t*) is distributed symmetrically around zero, giving rise to averaged descriptors of 0 and windowed descriptors for which the positive and negative versions have the same value. Conversely, for converged simulations of two *enantiomers*, the *abcdef*(*t*) distributions are each other’s opposite so that their averaged descriptors have the same values but opposite signs, and the positive and negative versions of the windowed descriptors are swapped.

## 3. Materials and methods

### 3.1. Conformational sampling

#### 3.1.1. Implicit solvent MD simulations.

The protocol by De Gauquier et al. [18] was used to generate a representative conformational ensemble of 43 chiral drugs, except that in addition to implicit water, the simulations were also performed using a GBMV (Generalized Born using Molecular Volume) implicit solvent model that was somewhat more representative for the experimental environment. For this purpose, the empirically fitted formula of Gagliardi et al. (equation (6) in [31]) was first used to determine that the relative dielectric constant of our 40/60 acetonitrile (ACN)/ water mixture at a temperature of 40 °C is approximately 59.3. As the authors of the GBMV model [32] have proposed a protocol to run GBMV simulations in solvents with arbitrary dielectric constants, this allowed us to tailor our solvent model to the ACN/water mixture used in the experiments. Specifically, we used the GBMV parameters obtained by using the value of 59.3 in equation (15) of reference [32]. The simulations were performed using CHARMM program “Free Version 46b1” (Git commit ID bde556660) [33]. Given that the conformational landscapes of two enantiomers are identical but mirrored, simulations were performed for only one enantiomer per pair. All test set molecules were constructed in their expected protonation state at the pH of the mobile phase (pH 9) as well as in an “uncharged state” where all protonatable groups are left in their neutral form, as a limiting case where the analyte is embedded in a highly nonpolar stationary phase. Then, force field representations of the test set molecules in their different states were obtained using CGenFF version 3.1 [23,34] and version 1.1.0 of the CGenFF program [24,27]. Subsequent simulations were performed using the Self-Guided Langevin Dynamics (SGLD) enhanced sampling method [35] in order to accelerate conformational transitions. Specifically, the most basic version of the SGLD method was performed using weighting factors to compute the weighted average chiral descriptor based on the canonical ensemble [36]. For each molecule in the test set, a 100 ns SGLD simulation at 313.15 K was run with 2 fs time steps (constraining bond lengths of H-atoms using the SHAKE algorithm [37]). A friction coefficient FBETa of 5 ps^-1^ and a guiding factor SGFT of 0.9 were set on non-hydrogen atoms (*γ* and *λ* in [36]). The Self-Guided Weight (SGWT) factors were set to 0 on the hydrogen atoms. The geometry of the molecule was sampled every 0.5 ps so that the chiral descriptors could be calculated on a total of 200,000 snapshots.

#### 3.1.2. Explicit solvent MD simulations.

GROMACS version 2018.1 [38] was used to perform all explicit solvent MD simulations of the different analytes both in their protonation states corresponding to pH = 9 and in their uncharged state. Each such state was placed in a cubic box that extended at least 1.0 nm from the edges of the molecule in all directions. Then, the remainder of the box was filled with a mixture of CGenFF acetonitrile and TIP3P water [39] molecules in 40/60, v/v proportions using the *gmx insert-molecules* and *gmx solvate* facilities in GROMACS. To alleviate bad contacts, an energy minimization was performed until the maximum force was below 1000.0 kJ/mol/nm. The equilibration consisted of a 100 ps NVT (number of particles (N), volume (V) and temperature (T)) run at 293.15 K with the Nose-Hoover thermostat [40], followed by another 100 ps of NPT (number of particles (N), pressure (P) and temperature (T)) at 1.0 bar using the Parrinello-Rahman barostat [41]. Finally, each MD simulation was performed for 100 ns with a 2 fs time step (constraining bond lengths of H-atoms using the LINCS algorithm [42]). A 1.2 nm cutoff was applied for long-range van der Waals energies and Coulomb interactions using the Verlet cutoff-scheme. The particle mesh Ewald method [43] was used for calculating long-range electrostatic interactions and the “dispcorr=enerpress” directive was included to compensate for the effect of the van der Waals cutoff on the energy and pressure [42]. For each MD simulation, a total of 10,000 snapshots were saved at regular intervals of 10 ps.

### 3.2. Vector and conformation-independent chiral descriptor calculation

First, the attributes in Table 1 were assigned to each atom based on its CGenFF atom type. This enabled the calculation of the 11 vectors listed in Table 1 and the 4 shape-based vectors described in §2.1b for each MD trajectory snapshot. Next, the triple product and shape-based conformation-dependent descriptors were calculated (S1 Table). For the “averaged” descriptors, the arithmetic mean of the per-frame conformation-dependent descriptor values was used for the explicit solvent simulations and the “unweighted” implicit solvent-based conformation-independent descriptors. Conversely, the “weighted” variant of the descriptors included the per-conformation SGLD weight factors [36] as calculated using the awk script from the SGLD chapter of the CHARMM documentation (discarding the first 200 samples, i.e., the first 100 ps, when calculating the descriptor’s weighted averages). The “windowed” versions of the descriptors were calculated according to Eq 7 and the SI, again applying the SGLD weight factors for the “weighted” variant. All steps in the present paragraph were performed in an automated fashion using in-house scripts.

The convergence of the descriptors was monitored; their values stabilized within 100 ns of simulation time for both implicit (SGLD) and explicit solvent simulations. As an illustration, the evolution of the averaged descriptor values of *ftwist*, *msagda* and *pihahb* is plotted in S2 Fig for the rigid molecule ibuprofen and the flexible molecule verapamil. Note that the descriptor data were standardized using Z-scores to facilitate comparison across different descriptors or systems.

### 3.3. Construction of QSER models

Retention factors *k* were calculated as *k* = (*t*_*R*_ – *t*_*0*_)/*t*_*0*_, with *t*_*R*_ the retention time of the corresponding compound and *t*_*0*_ the dead time.

Eight series of chiral descriptors (see Table 2) were considered for modelling. They included 5 series of descriptors for the molecules at their pH 9.0 protonation state: 1 set of chiral descriptors from the explicit solvent (water/ACN) simulations and 4 sets comprising both weighted and unweighted descriptors calculated from implicit solvent simulations with both solvent models (water and water/ACN). Conversely, the implicit solvent simulations of the molecules in their uncharged state were only performed using the water/ACN model, yielding a weighted and an unweighted set in addition to a 3^rd^ descriptor set based on the explicit solvent simulations (also in water/ACN).

**Table 2:** Overview of the 8 descriptor sets used for modelling.

Protonation state of the molecules	Solvent system considered for MD simulations	Descriptor calculation from MD ensembles
pH 9	Implicit water	Unweighted (I)
		Weighted (II)
	Implicit water/ACN	Unweighted (III)
		Weighted (IV)
	Explicit water/ACN	Unweighted (V)
Uncharged	Implicit water/ACN	Unweighted (VI)
		Weighted (VII)
	Explicit water/ACN	Unweighted (VIII)

The descriptor sets are labeled from I to VIII. Each set consists of a subset of averaged and windowed descriptors.

**Fig 4:**
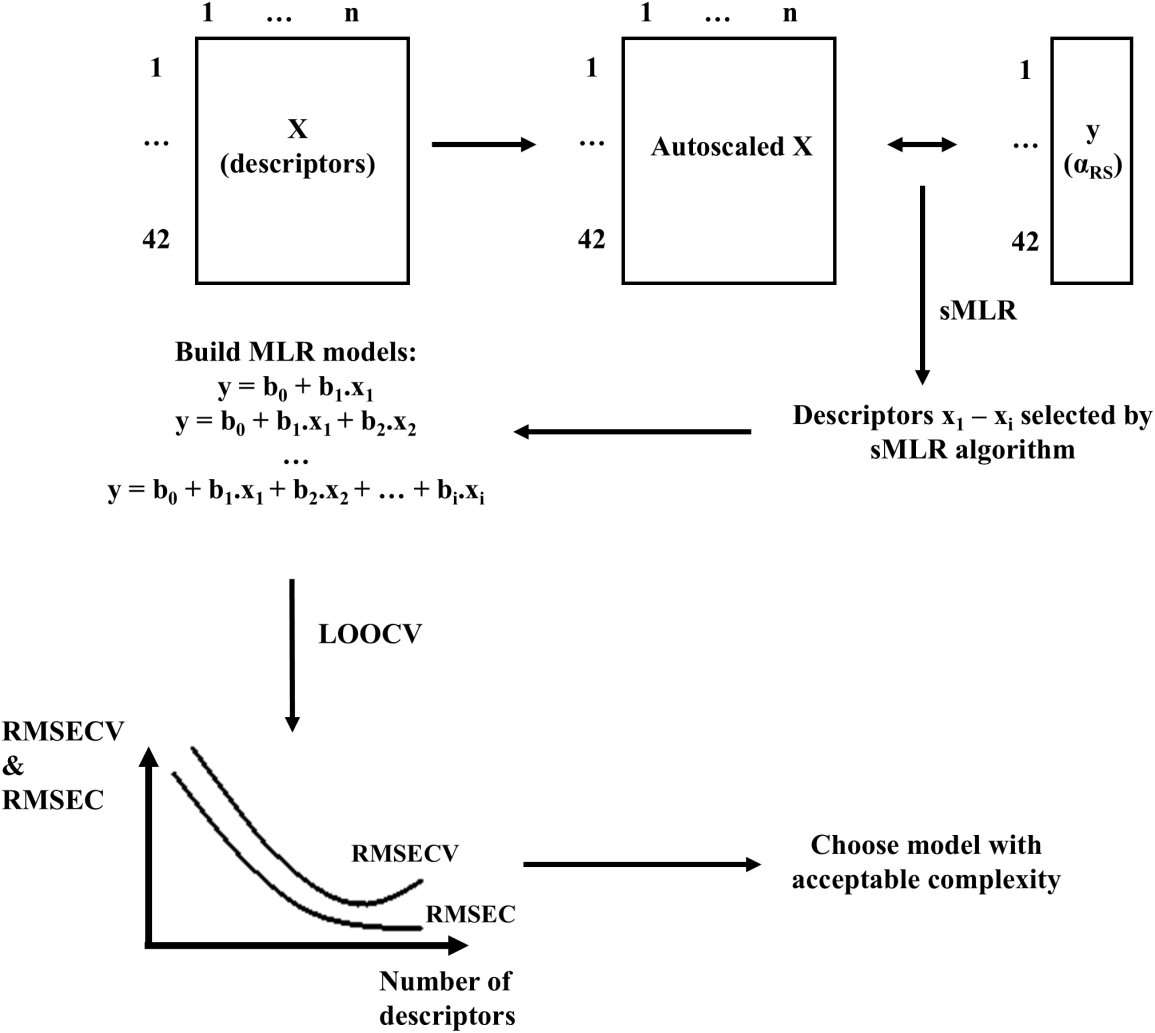
Overview of the different steps in QSER model building. Prior to model building, the descriptor values were autoscaled. As a result, the contributions of the descriptors to the model can directly be assessed by comparing their respective coefficients.

QSER models were built with only chiral descriptors as explanatory variables and an alternative selectivity factor, *α*_*RS*_, as response. The latter was obtained by dividing *k* of the R enantiomer by that of the S enantiomer, regardless of their actual elution order. Consequently, an *α*_*RS*_ above 1 means that the R enantiomer elutes last whereas a value below 1 implies that it elutes first. Since the same descriptor value with different signs is obtained for the two enantiomers when applying the averaged descriptors and the values for the negative and positive version are swapped for the two enantiomers when applying the windowed descriptors, the chiral descriptor values calculated on a single enantiomer can be used for modelling. Specifically, α_RS_ and log α_RS_ were linked to the chiral-descriptor values calculated on the R enantiomer. A detailed explanation of the modelling approach is provided below and illustrated in Fig 4.

MATLAB® 2021b (The Mathworks, Natick, MA, USA) was used to build stepwise multiple linear regression (sMLR) and partial least squares (PLS) regression models [44,45]. Both sMLR and PLS models with different complexities were built and the best model was determined from leave-one-out cross validation (LOOCV) results. LOOCV was chosen because the dataset was relatively small, and this approach ensured maximal use of the available data for model development.

In the sMLR algorithm, α was set by default to 5%. Where necessary, it was subsequently increased to be able to build more than 15 models, which was required to select the final model.

The complexity of selected models was determined as follow: initially, a graph was constructed of the root mean squared error of calibration (RMSEC) and root mean squared error of cross validation (RMSECV) as a function of the model complexity. Optimal complexity is found where RMSECV goes through a minimum (RMSECV_min_). However, often this is not the case and a behavior as for RMSEC (continuously decreasing tendency) is seen. Therefore, an alternative for the complexity at RMSECV_min_ has to be determined.

For this purpose, the relative change in RMSECV (ΔRMSECV) between consecutive models differing by one term, i.e., (RMSECV_A-1_ – RMSECV_A_)/RMSECV_A-1_, in which A represents the model complexity, was calculated. The minimal complexity for which ΔRMSECV becomes ≤ 0.02 or that of the bending point in the curve, is determined [46]. Then the average variance (RMSECV^2^_average_) of the subsequent 10 model complexities is calculated, as well as the variance RMSECV^2^_Crit_, which is not significantly larger than RMSECV^2^_average_, as determined by the following equation [47]:


$${\text{RMSECV}}_{\text{Crit}}^{\text{2}}=\;{\text{F}}_{\left(\alpha ,\text{ N},\text{ N}\right)}\;.\;{\text{RMSECV}}_{\text{average}}^{\text{2}}$$ (8)

with F(α, N, N) determined at α = 0.05 and N, N degrees of freedom (with N the number of compounds in the training set). The final model was then selected as the simplest model with an RMSECV^2^ not exceeding RMSECV^2^_Crit_.

For all selected models, RMSECV and RMSEC were calculated to validate the models. The RMSEC and RMSECV were normalized (represented as RMSEC_N_ and RMSECV_N_), by dividing by the experimental range of the response considered, to become comparable for models on different responses. In addition, the determination coefficient r^2^ between the experimental and predicted responses, and q^2^, the determination coefficient of the cross validation results, which can be related to the predictive abilities of the models, were determined. Subsequently, the average relative prediction error between the experimental and predicted responses was calculated to evaluate the model performance. For log *α*_*RS*_, the percentage average prediction error was backcalculated to *α*_*RS*_, i.e., calculating the absolute difference of the residues between the experimental and predicted *α*_*RS*_, divided by the experimental *α*_*RS*_ and multiplied by 100.

For log *α*_*RS*_ and *α*_*RS*_, the predicted versus experimental responses were plotted (log *α*_*RS*_ was first back calculated to *α*_*RS*_). Three types of prediction were evaluated. In a first instance, the prediction of the enantioselectivity is evaluated. The *α*_*RS*_ of a molecule was arbitrarily considered as accurately predicted when the experimental *α*_*RS*_ (further called *x*) and the predicted *α*_*RS*_ (further called *y*) do not differ by more than 0.05, thus *y* Є [x ± 0.05].

Secondly, it is evaluated whether the model correctly predicts that a molecule is separated or not. In this approach, a molecule was considered unseparated when *x* Є [0.95, 1.05], otherwise it is considered separated. When for a molecule *x* and *y* are both Є [0.95, 1.05], it is correctly predicted as unseparated. Otherwise, when *x* and *y* are both outside [0.95, 1.05], it is correctly predicted as separated.

Thirdly, the prediction of the elution sequence is evaluated, which was performed by dividing the above plot into four quadrants (See §4.1). The elution sequence was considered correctly predicted when x and y are both higher or lower than 1.0. Consequently, molecules that remained experimentally unresolved were excluded.

GraphPad Prism (GraphPad Software, San Diego, CA, USA, Version 10.0.0) was used to draw the plots. Dendrograms were created using the Scikit-learn Python package (version 1.6.0) [48].

### 3.4. k-Nearest Neighbours based applicability domain

An applicability domain was initially defined by applying the k-Nearest Neighbours (kNN) algorithm to the descriptor matrix. Specifically, the average distance between test set molecules and their k-nearest neighbours was calculated, and an overall average and standard deviation were determined to set a threshold. This threshold, calculated as the average plus twice the standard deviation, indicates whether a new molecule falls within the applicability domain of the model (with about 95% confidence) [49]. As distance measure, the Euclidian distance was used, and the value of k was set to 4 based on the empirical formula k = n^1/3^, where n represents the number of test set compounds [49]. All analyses were conducted in Matlab® 2021b.

### 3.5. Reagents

Methanol (MeOH) and ACN (VWR Chemicals, Leuven, Belgium) were HPLC grade. A 0.02 M borate buffer was prepared with boric acid (Merck, Darmstadt, Germany), of which the pH was adjusted to pH 9.0 with 1 M sodium hydroxide (Fisher Scientific, Pittsburgh, PA, USA). Ultrapure water was provided by an Arium Pro UV system (Sartorius Stedim Biotech, Göttingen, Germany). The buffer was vacuum-filtered through a 0.20 µm membrane (Sartorius Stedim Biotech), mixed with the organic modifier, and the mobile phase was degassed for 15 min in an ultrasonic bath (Branson, Brookfield, CT, USA) before use.

The test set consists of 40 racemates: acenocoumarol from Novartis (Basel, Switzerland), aminogluthetimide, baclofen, cetirizine, clopidogrel, equol, medetomidine and tipifarnib from Cayman Chemical (Ann Arbor, MI, USA), atenolol, etiracetam, ibuprofen, ketoprofen, mandelic acid, metalaxyl, ofloxacin, razoxane, sulpiride, tetramisole, tolterodine, verapamil and warfarin from Sigma Aldrich (St. Louis, MO, USA), blebbistatin and bupivacaine from Enzo (Farmingdale, NY, USA), fluoxetine from USP (Rockville, MD, USA), isoxanthohumol from HWI Group (Rülzheim, Germany), laudanosine, modafinil and tamsulosine (European Pharmacopoeia Reference Standards), lisofylline from Hoechst (Frankfurt am Main, Germany), omeprazole from RTC (Steinheim, Germany), propranolol from Certa (Braine l’Alleud, Belgium), carbinoxamine, indapamide, piperitone and praziquantel from TCI (Tokyo, Japan), ondansetron from Thermo Fisher Scientific, rolipram from Apollo Scientific (Stockport, UK), salbutamol from Glaxo Wellcome (London, United Kingdom), thalidomide from MP Biomedicals (Irvine, CA, USA), lansoprazole (gift from unknown origin). The minimum purity of these compounds was 94%. For pramipexole, carvone and rivaroxaban, no racemic mixture was available. Therefore, their R and S enantiomers were purchased.

Forty-six enantiopure compounds were analyzed besides the racemates to determine the elution sequence of the enantiomers: S(-)-acenocoumarol, R(+)-baclofen, R(+)-blebbistatin, S(-)-bupivacain, S(-)-isoxanthohumol, R(+)-lansoprazole, S(+)-laudanosine, R(-)-lisofylline, R(-)-modafinil, R(-)-salbutamol, R(+)-ofloxacine, S(-)-omeprazole, R(+)-pramipexole, R(-)-rolipram, S(-)-warfarine (Cayman Chemical), S(-)-aminogluthetimide, R(+)-atenolol, S(-)-equol, S(-)-etiracetam, R(-)-fluoxetine, S(+)-medetomidine, S(+)-metalaxyl, S(-)-pramipexole, S(-)-propranolol, S(+)-razoxane, S-rivaroxaban, S(-)-sulpiride, S(-)-tetramisole, R(+)-thalidomide, R(+)-tipifarnib, R(+)-tolterodine and S(-)-verapamil from Sigma Aldrich, S(+)-carvone and R(-)-carvone from Alfa Aesar, R(-)-cetirizine, R(-)-piperitone and R(-)-tamsulosine from TCI (Tokyo, Japan), S(+)-clopidogrel from Thermo Fisher Scientific, S(+)-ibuprofen and R(-)-mandelic acid from Acros Organics (Geel, Belgium), S(+)-ketoprofen from Enzo (Antwerp, Belgium) and R-rivaroxaban from USP. The minimum purity of these compounds was 94%. For carbinoxamine, indapamide, ondansetron and praziquantel, no enantiopure compound was available. These racemic mixtures were separated into their enantiomers by means of preparative supercritical fluid chromatography, in collaboration with Prof. E. Lipka, Department of Analytical Chemistry, University of Lille, France.

Racemates and enantiopure compounds of the test set were dissolved in MeOH at a concentration of 0.5 mg/mL. The samples were protected from light and kept in the fridge until analysis.

### 3.6. Chromatographic conditions

The analyses were performed on a LaChrom Elite HPLC system from VWR-Hitachi (Radnor, PA, USA), composed of an L-2200 autosampler, L-2350 column oven, L-2130 pump and L-2455 DAD detector. The separations were carried out in isocratic elution mode at a flow rate of 0.5 mL/min. The injection volume was 5 µL and the detection wavelength 220 nm. The column temperature was kept at 20 °C. The system was operated by EZChrom Elite software (version 3.3.2.SP2, VWR, 2017). The first disturbance of the baseline signal was used as the dead time.

The mobile phase consisted of 0.02 M borate buffer pH 9.0 and ACN in a 60/40 (V/V) ratio. A Lux amylose-2 CSP (250 x 4.6 mm i.d., 5 µm particle size) from Phenomenex (Torrance, CA, USA) was used. Under these conditions, mandelic acid was not retained and is therefore excluded from the model building and discussion.

## 4. Results and discussion

To summarize §3.1, conformational ensembles that are relevant for the mobile phase were generated by subjecting each of the analytes in its pH = 9 protonation state to three MD simulations: implicit water, implicit water/ACN and explicit water/ACN. Additionally, since the stationary phase environment is less favorable to net charges, the implicit and explicit water/ACN simulations were also performed with uncharged protonatable groups. All implicit solvent simulations were performed using a variant of the SGLD enhanced sampling method that produces weight factors to correct for the bias in the conformational ensemble introduced by its enhancement in sampling. However, questions about the robustness of these weight factors have been raised [50], prompting us to generate sets of ensemble-averaged descriptors with and without using said weight factors. As explained in §3.3 (Table 2), this gave rise to a total of eight sets of (averaged and windowed) conformation-independent descriptors. Accordingly, the methodology for building QSER models with different sets of descriptors (Table 2) is discussed in the next section. Each of these sets contains a subset of 167 averaged and a second subset of 334 windowed descriptors, as explained at the end of the introduction.

### 4.1. QSER models of *α*_*RS*_ and log *α*_*RS*_

The experimentally determined *t*_*R*_, *k, α*_*RS*_ and log *α*_*RS*_ values from the analysis of the test set compounds on Lux amylose-2 with the basic mobile phase are given in S2 Table. For each racemic mixture, the enantiomeric elution sequence was determined with the corresponding enantiopure compound. The aforementioned eight sets of descriptors (sets I – VIII in Table 2) were applied individually and combined as explanatory variables to build sMLR and PLS models for log *α*_*RS*_ and *α*_*RS*_. These models can be found in S3–S4 Tables. As the PLS models do not perform better than the sMLR models, we will limit ourselves to discussing the latter.

In a first instance, descriptor sets I – V (Table 2) were applied individually and together to build models (Eqs S3 – S12), with the averaged and windowed descriptors in sets III, IV and V also being evaluated separately, allowing us to investigate the influence of the implicit and explicit solvent descriptors on the modelled response. No significant difference was observed between the models based on implicit water and implicit water/ACN descriptors. However, the model with explicit solvent descriptors (Eq S7) show better performance parameters and predictions than those with implicit solvent descriptors (Eqs S3-S4). The observation that a more realistic solvent model appears to yield better separation models might be an indication that the conformation-dependent descriptors as defined in this paper exhibit a physically meaningful correlation with the separation. Additionally, heat maps were made to compare the correlations between sets of descriptors (see S5 Table for which descriptor corresponds to which number). From the heat map in S3 Fig, it can be inferred that the descriptors from the implicit water simulations are strongly correlated with their implicit water/ACN counterparts (S3A Fig), while the correlation was somewhat lower between implicit and explicit solvent descriptors (S3B Fig). Considering the solvent system used in the chromatographic experiments, the explicit and implicit water/ACN descriptors were retained in our QSE(R)R modelling. Furthermore, the model’s performance was examined using either averaged or windowed descriptors, specifically focusing on their effects when applying unweighted or weighted implicit solvent descriptors. The models built with the weighted descriptors (Eqs S9-S10) showed slightly better performance compared to those using the unweighted descriptors (Eqs S11-S12).

Subsequently, the averaged and windowed descriptors from sets VII and VIII (Table 2) were applied both individually and together (Eqs S13-S15) to evaluate their performance in models developed for predicting log *α*_*RS*_. Additionally, the averaged and windowed descriptors from sets VI and VIII (Table 2) were used together (Eq S16) to compare the model’s performance when employing either weighted or unweighted implicit solvent descriptors. This analysis also aimed to determine whether model improvement may be observed when applying the descriptors calculated from the uncharged molecules. The best model (Eq S14) was based on 9 descriptors (6 from set VIII and 3 from set VII) and predicts log αRS accurately for 22/42 molecules. However, it performs only slightly better than the model with both averaged and windowed descriptors (Eq S15). Notably, this model contains only one averaged descriptor and is less complex. Because this model performs best, it highlights the significance of chiral descriptors derived from uncharged molecules. This can be justified by the fact that the analyte exists in an equilibrium between its protonated and unprotonated forms when moving through the chromatographic system, where the latter form will preferably interact with the selector.

**Fig 5:**
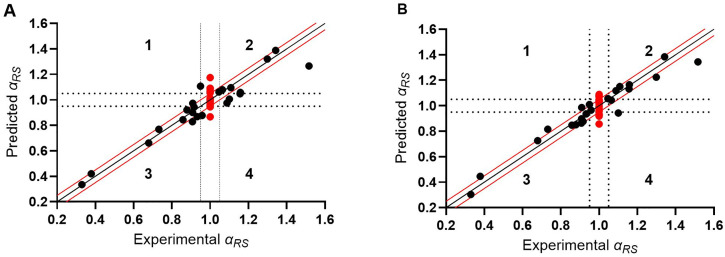
sMLR models: predicted *α*_*RS*_ as a function of the experimental one. **Used descriptors: sets IV, V, VII and VIII (Table 2).** Modelled responses: (A) log *α*_*RS*_, and (B) *α*_*RS*_. The dashed lines divide the graph into four quadrants (indicated by numbers 1, 2, 3 and 4) and the black full line is the bisector. The red lines are the limits for what is considered an accurate *α*_*RS*_ prediction. Forty-two molecules were involved in the modelling and the red dots correspond to the experimentally unseparated molecules.

Finally, descriptor sets III – VIII were applied together, leading to models (Eqs 9–12 in Table 3) with better performance parameters than before. Here, the “averaged” and “windowed” schemes for calculating conformation-independent descriptors from a time series of conformation-dependent descriptor values (as explained in the introduction) are compared based on 4 models. In model A (Eq 9 in Table 3), log *α*_*RS*_ was modelled using four sets (IV, V, VII and VIII) of averaged descriptors as explanatory variables. Model B (Eq 10) used the same four sets of windowed descriptors. Models C and D (Eqs 11 and 12) used a combination of the four sets (IV, V, VII and VIII) averaged and windowed descriptors, modelled for log *α*_*RS*_ and *α*_*RS*_, respectively. For models C and D, plots of the predicted *α*_*RS*_ as a function of the experimental are given in Fig 5.

**Table 3:** Overview of models built from four descriptor sets (IV, V, VII and VIII given in Table 2), their performance parameters (see §3.3) and equations.

Parameter	Model A	Model B	Model C	Model D
	*Averaged*	*Windowed*	*Averaged + windowed*	*Averaged + windowed*
**RMSEC**_**N**_	0.0828	0.0351	0.0488	0.0533
**RMSECV**_**N**_	0.123	0.0747	0.0656	0.0814
**r** ^**2**^	0.76	0.96	0.92	0.90
**q** ^**2**^	0.29	0.87	0.73	0.84
**Number of descriptors**	7	11	8	7
**Prediction error (%)**	9.95	4.07	5.89	5.32
**Accurate predictions**	15/42	29/42	22/42	27/42
**Correct predictions**	23/42	32/42	26/42	30/42
**Elution sequence**	17/23	21/23	21/23	21/23
Model A: Log *α*_*RS*_ = −0.020–0.019 *gspiha (explicit uncharged)* – 0.099 *msachd (explicit uncharged)* + 0.12 *msachd (implicit uncharged)* + 0.079 *mschhd (implicit uncharged)* + 0.032 *stwist (explicit uncharged)* – 0.021 *gshahb (explicit uncharged)* – 0.059 *acalda (implicit charged)* (9)				
Model B: Log *α*_*RS*_ = −0.020–0.074 *msgshb- (explicit uncharged)* + 0.057 *agsiso+ (explicit uncharged)* – 0.035 *achdda- (implicit charged)* – 0.042 *gsalhb- (explicit uncharged)* – 0.035 *mschag+ (implicit uncharged)* – 0.021 *gspihb+ (explicit uncharged)* - 0.041 *acsiso- (explicit charged)* + 0.038 *chagpi- (implicit uncharged)* – 0.034 *acgspi+ (explicit uncharged)* + 0.032 *hasiso- (explicit charged)* - 0.015 *msfifo+ (implicit charged)* (10)				
Model C: Log *α*_*RS*_ = −0.020–0.075 *msgshb- (explicit uncharged)* + 0.054 *agsiso+ (explicit uncharged)* – 0.097 *achdda- (implicit charged)* – 0.037 *gsalhb- (explicit uncharged)* – 0.053 *mschag (implicit uncharged)* + 0.059 *gsaghd- (explicit charged)* + 0.039 *acgshb- (implicit charged)* – 0.024 *acgspi+ (explicit uncharged)* (11)				
Model D: *α*_*RS*_ = 0.98 + 0.12 *agsiso+ (explicit uncharged)* – 0.11 *achdda- (implicit charged)* – 0.11 *gsalhb- (explicit uncharged)* – 0.10 *mschag (implicit uncharged)* – 0.10 *msgsag- (explicit uncharged)* + 0.085 *mschhb+ (implicit uncharged)* – 0.061 *agalha+ (implicit charged)* (12)				

Model A: log *α*_*RS*_ modelled using only the averaged descriptors from sets IV, V, VII and VIII. Model B: log *α*_*RS*_ modelled using only windowed descriptors from sets IV, V, VII and VIII. Model C: log *α*_*RS*_ modelled using the combination of averaged and windowed descriptors from sets IV, V, VII and VIII.

Model D: *α*_*RS*_ modelled using the combination of averaged and windowed descriptors from sets IV, V, VII and VIII.

A somewhat better model appears to be obtained when the sMLR algorithm is applied to a broader selection of descriptor sets. This is observed when comparing models A and B, because nearly all performance parameters improved. Conversely, when the sMLR algorithm was provided with all averaged and windowed descriptor sets to build model C, a significant improvement in RMSECV_N_ (but not in the other performance parameters) is seen in comparison with model B, thus indicating that model C would yield similar prediction errors but is more robust with regard to different selections of test set compounds. When we take a closer look at the selected descriptors in model C, it is observed that seven out of eight are from the windowed set. The dominance of the windowed descriptors in these models can be justified by the use of window functions. For the sake of argument, let us consider a single bar *j* in the histogram *H(x)* of the scaled conformation dependent descriptor x given Fig 3. The width *w* of this bar in *x* represents a (small) range in descriptor values, corresponding to the subset *j* of analyte conformations for which the descriptor value falls within this range. Let us now define a “constrained binding constant” with the chiral stationary phase *K*_*i,j*_ *for only the conformational (sub-)ensemble j*. Assuming that the histogram is normalized, we also know that sub-ensemble *j* represents a fraction of the total conformational ensemble in solution proportional with *H*_*j*_: the height of bar *j*. This yields the following expression for the unconstrained macroscopic binding constant *K*_*i*_ for the full solution-phase conformational ensemble:


$${\;K}_{i}=w\sum_{j=\text{1}}^{m}K_{i,j}H_{j}=\int_{-\text{1}}^{\text{1}}K_{i}(x)H(x)dx$$ (13)

where the function *K*_*i*_(*x*) is the continuous counterpart of the hitherto discrete variable *K*_*i,j*_. Doing so reveals a strong analogy with the integral in Eq 7, wherein we try to obtain descriptors *abcdef±* that are linearly correlated with binding constants *K*_*i*_^*±*^ (associated with the retention factors of the R-enantiomer (*k*_*R*_) and the S-enantiomer (*k*_*S*_)) and where *f* ^±^(*x*) functions as a proxy for *K*_*i*_(*x*) or *K*_*i,j*_.

In pilot studies with smaller test sets and only averaged descriptors, modelling log *α*_*RS*_ resulted in better fitting models (results not shown). This was in line with the fact that log *α*_*RS*_ is directly linked with the difference in binding free energy between the enantiomers (Eq 1), which was assumed to correlate linearly with the averaged descriptor values (as also hinted by the fact that both quantities retain their values but switch signs when swapping enantiomers). However, when a model D was built for *α*_*RS*_ instead of log *α*_*RS*_, the performance parameters further improved compared to models B and C. This can be explained by the dominance of the windowed descriptors in these models which as rationalized above would be expected to correlate with (ratios of) binding constants rather than (differences in) binding free energies, and thus with *α*_*RS*_ rather than log *α*_*RS*_.

In the final model, 27 out of 42 *α*_*RS*_ (Fig 5B) values were predicted in the range of *α*_*RS,experimental*_ ± 0.05. Concerning the weighted and unweighted implicit solvent descriptors, heat maps were constructed (S4 Fig). Since these sets were strongly correlated, either the weighted or unweighted implicit solvent descriptors should be retained in our QSER models, but not both. S6 Table and S5 Fig present the performance parameters and plots, respectively, when modelling with the unweighted implicit solvent descriptors. When comparing S6 Table in the SI to the performance parameters using the weighted descriptors (see Table 3: models C and D), it is observed that the latter perform better. Specifically, the RMSEC_N_ and RMSECV_N_ obtained with the weighted implicit solvent descriptors are lower, and the r^2^ and q^2^ are closer to 1.0, indicating they show a better fit and predictive abilities. This is substantiated by the better prediction of the enantiomeric separation and the enantioselectivity.

Since the windowed descriptors from the explicit solvent simulations (sets V and VIII) are particularly prominent in our models, we tried using only these descriptors to build models for both log *α*_*RS*_ (Eq S19 and S6A Fig) and *α*_*RS*_ (Eq S20 and S6B Fig). The model for *α*_*RS*_ showed better performance parameters and predictive abilities compared to the log *α*_*RS*_ model (S7 Table), but overall, the models with wider descriptor sets discussed above performed even better.

Models C and D (Table 3) exhibited the highest performance of all models evaluated in this study. They show the best performance parameters and provided reliable predictions regarding elution sequence, separation and enantioselectivity.

An applicability domain for the test set was established based on the chiral descriptors (averaged and windowed descriptors from sets IV, V, VII and VIII in Table 2) retained in the final models (models C and D in Table 3). This was initially done using the kNN algorithm as detailed in §3.4; the results and threshold are provided in S2 File. Additionally, principal component analysis (PCA) and robust PCA, following Hubert et al. [51], were also used to define the applicability domain. According to the kNN-based threshold, two molecules (tipifarnib and cetirizine) fell outside the domain or were borderline. PCA identified three (tipifarnib, clopidogrel and cetirizine), and robust PCA four (tipifarnib, clopidogrel, cetirizine and blebbistatine). However, these molecules were not removed from the test set, because excluding them did not improve the model performance compared to model D (Table 3 versus SI) and neither could their outlying character be substantiated with experimental or structural arguments. However, the descriptor vectors of new molecules can be tested against the applicability domain to verify the validity of their predictions.

In conclusion, satisfactory models were obtained for the *α*_*RS*_ of a chemically diverse set of analytes by applying the averaged and windowed descriptors calculated in the native protonation state in the mobile phase as well as the uncharged state. Such models may potentially be useful for the prediction of the enantioselectivity, separation and elution sequence of chiral molecules.

### 4.2. Evaluation of the chiral descriptors

#### 4.2.1. Distribution of chiral descriptor values.

**Fig 6:**
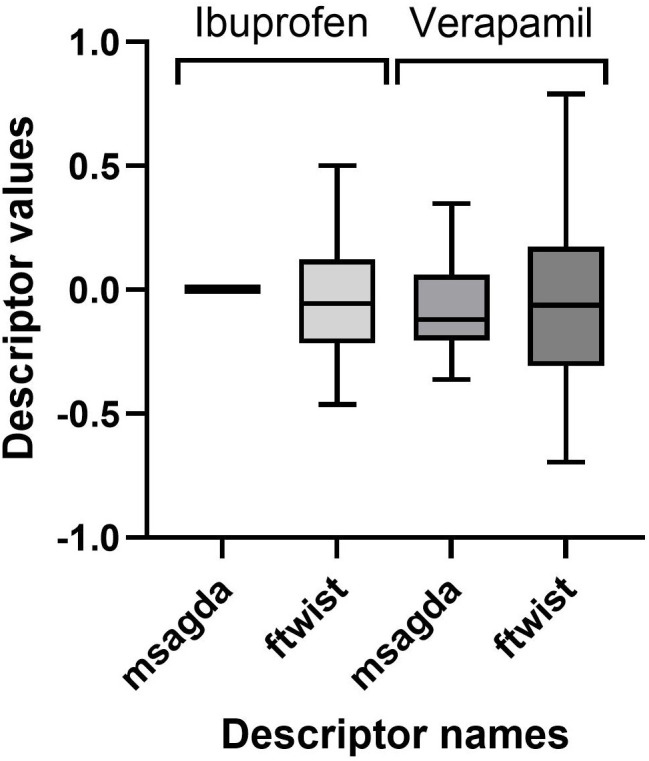
Chiral descriptor value box plots for *msagda* (see Table 1) and *ftwist* (see §2.1b). The descriptors are obtained from MD simulations in explicit water/ACN (10,000 snapshots), for ibuprofen and verapamil.

The averaged chiral descriptors calculated from MD simulations in implicit water, implicit water/ACN and explicit water/ACN were compared for two molecules with a different conformational flexibility, i.e., ibuprofen and verapamil (Fig 6). As representative examples, the descriptors *msagda* and *ftwist* were selected because they are present in some models, indicating their importance for predicting the enantioselectivity, and because *msagda* is a triple product descriptor while *ftwist* is a twist descriptor. For all solvent models, the distribution of the descriptor values is wider for verapamil than for ibuprofen, in line with the former’s higher conformational flexibility.

#### 4.2.2. Correlation between chiral descriptor values.

In this section, the correlation between implicit and/or explicit (averaged and/or windowed) solvent descriptors was plotted to assess whether complementarity exists between the descriptors. Strong correlations between descriptors can negatively impact modelling. Therefore, it is important to identify which descriptor sets are highly correlated.

To visualize the correlation between the averaged chiral descriptors, 2D heat maps of their correlation coefficients r were constructed. In addition, a hierarchical (complete linkage) clustering was performed using (1 - |r|) as a distance function, giving rise to dendrograms. In S7 Fig, the heatmaps are shown from the averaged chiral descriptors calculated from the molecules at their pH 9.0 state simulated in implicit water/ACN (S7A Fig) and explicit water/ACN (S7B Fig). The lower amount of yellow in the former plot suggests that the implicit solvent descriptors are slightly more diverse, which is confirmed by the higher number of branches below 1 - |r| < 0.2 in dendrogram S9 Fig compared to S8 Fig, and is explained by the use of an enhanced sampling method (SGLD) in the implicit solvent simulations, which gave rise to higher conformational diversity. Upon closer examination of the dendrograms, it was observed that chiral descriptors containing at least one HB vector and at least one non-HB vector (e.g. ${𝐯}_{𝐠𝐬}$), often appear together at (1 - |r| < 0.2), thus showing a high correlation (see S5 Table for which descriptor corresponds to which number).

Subsequently, the bisecting line on a correlation heat map for the chiral descriptors (implicit versus explicit) of uncharged chiral molecules (S10 Fig) shows that descriptors from implicit solvent simulations are highly correlated with their explicit-solvent counterparts. This aligns with the observation that Eq S9 (including both implicit and explicit solvent descriptors) has predictive abilities similar to Eq S7 (only explicit solvent descriptors).

**Fig 7:**
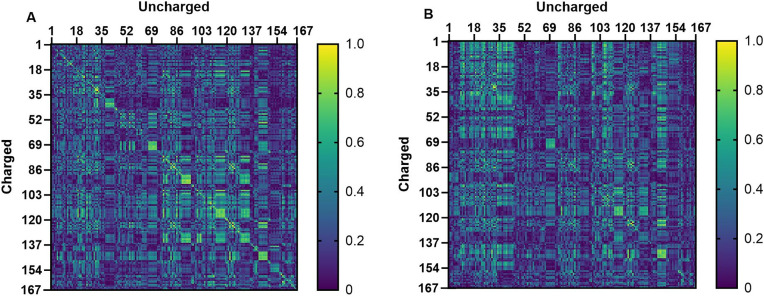
Heat maps from the correlation coefficients calculated between the averaged chiral descriptors obtained from different MD simulations from the test set molecules in their actual state at pH 9 and their uncharged state. The correlation is calculated between descriptors from charged and uncharged molecules in (A) implicit water/ACN and (B) explicit water/ACN. Numbers 1–167: number of a chiral descriptor, given in S5 Table.

Finally, heat maps for chiral descriptors from both the molecule’s native protonation state in the mobile phase and its uncharged state show modest correlation in implicit solvent (Fig 7A) and almost none in explicit solvent (Fig 7B). This demonstrates the strong impact of the protonation state on a molecule’s conformational preference and noncovalent interaction profile. In addition, it might suggest a shortcoming of the implicit solvent model to fully capture this impact, although the difference between the implicit and explicit solvent simulations might alternatively be due to the use of the SGLD enhanced sampling method in the former. Either way, it can be concluded that descriptors obtained from simulations on (de)protonated (charged) and uncharged molecules differ substantially.

As explained in the introduction, the windowed descriptors come in pairs consisting of a negatively and a positively windowed version, which are directly related to the amount of sampling of negative and positive values of the respective conformation-dependent descriptor during the simulation. S11 Fig indicates a high correlation when comparing both versions for the same MD simulation approach; it would appear that high values of the (+)-windowed descriptor correspond to low values of its (-)-windowed counterpart and vice versa. Consequently, *for the purpose of assessing the correlations between the averaged and windowed descriptors*, it suffices to only consider their (-)-windowed versions. Examining the correlation between these (-)-windowed descriptors resulting from simulations of (de)protonated analytes yields similar heat maps and the same conclusions as for the averaged descriptors: the protonation state makes a substantial difference, and this seems somewhat more pronounced in explicit solvent (though the latter difference is smaller than for the averaged descriptors). Finally, the correlation is compared between the windowed and averaged chiral descriptors (S12 Fig). When comparing these descriptors calculated from the same simulation, they show a high similarity (yellow-colored diagonal).

In summary, the descriptors calculated from implicit water and implicit water/ACN MD simulations show a high correlation, while they show a lower correlation with those calculated from explicit solvent simulations. Furthermore, it was observed that the windowed descriptors appear to perform better than the averaged ones if only one approach is included in the models. However, they show complementarity: the best models were obtained using both types of descriptors simultaneously. In addition, descriptors obtained from molecules in their uncharged state are important for the modelling and show complementarity with the descriptors derived from molecules in their pH 9 state. Finally, it was observed that the implicit solvent descriptors calculated using the SGLD weight factors yielded somewhat better models than their unweighted counterparts. Combining all these observations, we expect and see that the best performing models would be obtained by using the averaged and windowed descriptors from molecules in both their charged and uncharged state calculated from implicit and explicit MD simulations in a mixture of water/ACN.

It is apparent from Table 3 that the conformation-dependent descriptors *agsiso*, *achdda*, *gsalhb* and *mschag* occur with prominent coefficients in all three models B, C, and D, which supports the consistent nature of our modeling methodology. To further interpret the models from Table 3 in terms of the selected descriptors, it is helpful to divide the vectors that constitute the triple product descriptors into the following rough categories:

- **ms**, **al**: related to strength of London Dispersion force;
- **ch**, **gs**, **da**: related to (signed) charge;
- **ac**, **ag**, **hb**: related to hydrophilicity (regardless of positive or negative charge).

(Note that **pi**, **hd**, **ha**, siso and fifo are left uncategorized because they are not as closely related to other properties.) In the entirety of the final models, only 1 descriptor was selected that contained two vectors in the same category (*acgshb-*, containing both **ac** and **hb**, in Model C, albeit with a relatively low coefficient). This further supports the idea that the modeling methodology gravitates toward meaningful triple products (which combine significantly different vectors) and also opens the future perspective of more aggressively eliminating descriptors containing “similar” vectors (which we are currently doing only for [**ch**, **gs**], [**ac**, **ag**] and a few particularly redundant combinations of properties related to hydrogen bonds).

## 5. Conclusions

501 simulation-based “ESEC” descriptors representing physically meaningful chiral properties of arbitrary solutes were defined and made publicly available (see “Data Availability Statement”). For the purpose of validation, QSERR models were built for predicting the enantioselectivity of a structurally diverse set of pharmaceuticals on a polysaccharide-based stationary phase. An effort was made to elucidate the relative importance of the different subclasses of descriptors (implicit vs. explicit and averaged vs. windowed) for the purpose of predicting enantioseparation. Specifically, sMLR models were built with the chiral descriptors derived from MD simulations using implicit water, implicit water/ACN and explicit water/ACN solvent models for molecules protonated at pH 9. Descriptors derived from these simulations show a high correlation; however, the models with explicit solvent descriptors performed better. This may indicate the importance of specific solvent interactions during the MD simulations in obtaining a relevant conformational ensemble.

In addition, windowed and averaged ensemble averaging methods were compared. It was shown that the descriptors from both types can be complementary, but the windowed chiral descriptors globally performed best. This is in agreement with the chiral recognition model on which the present work is based.

Finally, including descriptors derived from simulations of compounds in their uncharged state provided a significant improvement of the QSER model. This agrees with a hypothetical recognition process in which the analytes lose their pH-induced charges upon migration to the (globally apolar) chiral stationary phase and bind to the chiral selector in their neutral form. The best model in the present study uses *α*_*RS*_ as response and showed a leave-one-out cross validation error of 0.0814, a well-predicted elution sequence for 21 out of 23 separated chiral molecules and accurate enantioselectivity predictions for 27 out of 42 chiral molecules. Out of the 7 descriptors in this model, the majority consisted of windowed descriptors calculated on uncharged molecules in an implicit and explicit solvent.

Taken together, our results seem to indicate that simulations in a realistic environment are important to obtain physically meaningful chiral descriptors that correlate with the chiral interactions in the presently considered chromatographic system. As such, the present work is an incremental step toward solving the “grand challenge” of predicting the enantioselectivity and elution sequence of arbitrary chiral molecules for a given chromatographic system. Future work includes optimizing the window functions of the windowed descriptors, eliminating statistically redundant descriptors prior to modelling, and constructing models for a wider range of chromatographic systems.

## Supporting information

S1 File Derivation of the used window functions for the windowed window functions. (DOCX)

S2 File Applicability domain. (DOCX)

S1 Fig Examples of atropisomers. (DOCX)

S2 Fig Running means of averaged descriptors ftwist, msagda and pihahb for ibuprofen (left) and verapamil (right). (DOCX)

S3 Fig Heat map for the correlation coefficients calculated between the averaged chiral descriptors obtained from different MD simulations. (DOCX)

S4 Fig Heat map for the correlation coefficients calculated between averaged chiral descriptors obtained from different MD simulations for the test set molecules. (DOCX)

S5 Fig sMLR models: predicted αRS as a function of the experimental. (DOCX)

S6 Fig sMLR models: predicted αRS as a function of the experimental. (DOCX)

S7 Fig Heat maps from the correlation coefficients calculated between the averaged chiral descriptors obtained from MD simulations from the test set molecules in their actual state at pH 9. (DOCX)

S8 Fig Dendrogram from the correlation coefficients calculated between the averaged chiral descriptors obtained from implicit water/ACN simulations. (DOCX)

S9 Fig Dendrogram from the correlation coefficients calculated between the averaged chiral descriptors obtained from explicit water/ACN simulations. (DOCX)

S10 Fig Heat map for the correlation coefficients calculated between the averaged chiral descriptors obtained from different MD simulations from the test set molecules in their uncharged state. (DOCX)

S11 Fig Heat maps for the correlation coefficients calculated between the negatively (-) and positively (+) windowed chiral descriptors obtained from different MD simulations from the test set molecules in their charged state. (DOCX)

S12 Fig Heat maps for the correlation coefficients calculated between the negatively (-) windowed chiral descriptors obtained from MD simulations from the test set molecules in their charged and uncharged state. (DOCX)

S1 Table Averaged chiral descriptors for R-medetomidine, protonated at pH 9 and simulated in implicit water/ACN. (DOCX)

S2 Table Chromatographic results with 0.02 M borate buffer pH 9/ ACN (60/40 V/V) on Lux amylose-2. (DOCX)

S3 Table Overview of sMLR models for enantioselectivity, built with different types and combinations of chiral descriptors. (DOCX)

S4 Table Overview of PLS models for enantioselectivity, built with different types and combinations of chiral descriptors. (DOCX)

S5 Table Number related to a given averaged chiral descriptor. (DOCX)

S6 Table sMLR models built with different types and combinations of unweighted chiral descriptors (sets III, V, VI and VIII). (DOCX)

S7 Table sMLR models for αRS and log αRS using only the windowed descriptors acquired from explicit solvent MD simulations. (DOCX)

## Abbreviations

ACN: acetonitrile
CGenFF: Charmm General Force Field
CSP: chiral stationary phase
ESEC: Ensemble Steric and Electrostatic Chirality
GBMV: generalized Born using molecular volume
HB: hydrogen bonds
HBA: hydrogen bond acceptor
HBD: hydrogen bond donor
HPLC: high-performance liquid chromatography
kNN: k-Nearest Neighbours
LOOCV: leave-one-out cross validation
MD: Molecular Dynamics
MeOH: methanol
NPT: number of particles (N), pressure (P) and temperature (T)
NVT: number of particles (N), volume (V) and temperature (T)
PCA: principal component analysis
PLS: partial least squares regression
QSAR: Quantitative Structure-Activity relationship
QSER: Quantitative Structure-Enantioselectivity Relationship
QSERR: Quantitative Structure-Enantioselective Retention Relationship
QSRR: Quantitative Structure-Retention Relationship
RMSEC: root mean squared error of calibration
RMSECV: root mean squared error of cross validation
RMSECV_min_: minimal root mean squared error of cross validation
RPLC: reversed-phase liquid chromatography
SEM: Standard Error on the Mean
SGLD: Self-Guided Langevin Dynamics
sMLR: stepwise multiple linear regression
