# Supplementary material for: Development of Ensemble Steric and Electrostatic Chirality (ESEC) descriptors for modelling chromatographic enantioseparations
Source: PLoS One. 2025 Oct 17;20(10):e0333635. doi: 10.1371/journal.pone.0333635 (PMC12533851; doi:10.1371/journal.pone.0333635)
Supplement: S1 File — (DOCX) [file pone.0333635.s001.docx]

**Derivation of the used window functions for the windowed window functions**

For the derivation of the window functions we opted for a sum of cosines because this class of functions has favorable mathematical properties; specifically, its members reach 0 at a given, finite distance. Also, the sum of two such functions is again a sum of cosines (albeit with different phases); while this is of no direct utility in the present, it might be in future research. Thus, we took the 4-term Nuttall window function [1] and algorithmically reoptimized the coefficients *a_i_* (to a precision of 10^-9^) to minimize its overlap with the parabola *x*^2^ (Eq S1)

$$\begin{aligned} f^{0}\left( x \right)=a_{0}+a_{1}\cos({2\pi x}/W)+a_{2}\cos({4\pi x}/W)+a_{3}\cos({6\pi x}/W) \\ \text{with }\left( a_{0},a_{1},a_{2},a_{3} \right)=\underset{a_{0},a_{1},a_{2}=0.5-a_{0},a_{3}=0.5-a_{1}}{\arg\min} \int_{0}^{1} f_{W=2}^{0}\left( x \right)x^{2}dx \#\left( \text{S}\text{1} \right) \end{aligned}$$

subject to the constraint that *f* ^0^(*x*) *retains exactly one inflection point* in the domain [0,1] for width *W* = 2. This minimization has the effect of further narrowing the peak of the function. Using the resulting coefficients (0.302375557, 0.461344709, 0.197624443, 0.038655291), the final window functions *f* ^±^ in Eq S2 were generated by setting the width *W* = 3 and offsetting x by ±0.5, i.e.

$$f^{+}\left( x \right)= f_{W=3}^{0}\left( x-0.5 \right)\text{ and }$$

$$\begin{aligned} f^{-}\left( x \right)= f_{W=3}^{0}\left( x+0.5 \right)\#\left( \text{S}\text{2} \right) \end{aligned}$$

[1] Nuttall A. Some windows with very good sidelobe behavior. IEEE Trans. Signal Process. 1981 Feb; 29(1):84-91. Available from: https://doi.org/10.1109/TASSP.1981.1163506
