# Supplementary material for: Development of Ensemble Steric and Electrostatic Chirality (ESEC) descriptors for modelling chromatographic enantioseparations
Source: PLoS One. 2025 Oct 17;20(10):e0333635. doi: 10.1371/journal.pone.0333635 (PMC12533851; doi:10.1371/journal.pone.0333635)
Supplement: S2 File — (DOCX) [file pone.0333635.s002.docx]

**Applicability domain**

**1) k-Nearest Neighbours**

The k-Nearest Neighbours (kNN) algorithm, with k = 4, was applied to descriptor sets IV, V, VII and VIII from Table 2, yielding the following results:

- Overall average Euclidean distance = 43.0

- Standard deviation = 16.7

- Threshold: average + (2 x standard deviation) = 76.4

**2) Models after removing potential outliers**

Model for *α_RS_* without cetirizine and tipifarnib using a combination of the four sets (IV, V, VII and VIII in Table 2) averaged and windowed descriptors:

The final model has 7 descriptors and the following performance parameters:

- RMSECV_N_ = 0.104; RMSEC_N_ = 0.0571; r^2^ = 0.89; q^2^ = 0.82; prediction error: 5.90%

- Accurate predictions: 22/40; correct predictions: 27/40; elution sequence: 21/22

Model for *α_RS_* without cetirizine, clopidogrel and tipifarnib using a combination of the four sets (IV, V, VII and VIII in Table 2) averaged and windowed descriptors:

The final model has 9 descriptors and the following performance parameters:

- RMSECV_N_ = 0.0884; RMSEC_N_ = 0.0497; r^2^ = 0.91; q^2^ = 0.83; prediction error: 4.93%

- Accurate predictions: 23/39; correct predictions: 29/39; elution sequence: 21/22
