# Supplementary material for: Development of Ensemble Steric and Electrostatic Chirality (ESEC) descriptors for modelling chromatographic enantioseparations
Source: PLoS One. 2025 Oct 17;20(10):e0333635. doi: 10.1371/journal.pone.0333635 (PMC12533851; doi:10.1371/journal.pone.0333635)
Supplement: S1 Fig — (DOCX) [file pone.0333635.s003.docx]

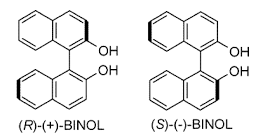

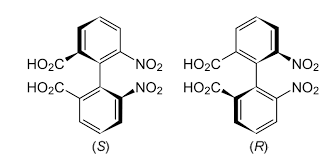


**S1 Fig.** **Examples of atropisomers.**

Left: atropisomer pairs of 6,6'-dinitro-2,2'-diphenic acid. Right: atropisomer pairs of 1,1′-binaphthyl-2,2′-diol (BINOL).
