# Supplementary material for: Development of Ensemble Steric and Electrostatic Chirality (ESEC) descriptors for modelling chromatographic enantioseparations
Source: PLoS One. 2025 Oct 17;20(10):e0333635. doi: 10.1371/journal.pone.0333635 (PMC12533851; doi:10.1371/journal.pone.0333635)
Supplement: S2 Fig — (DOCX) [file pone.0333635.s004.docx]

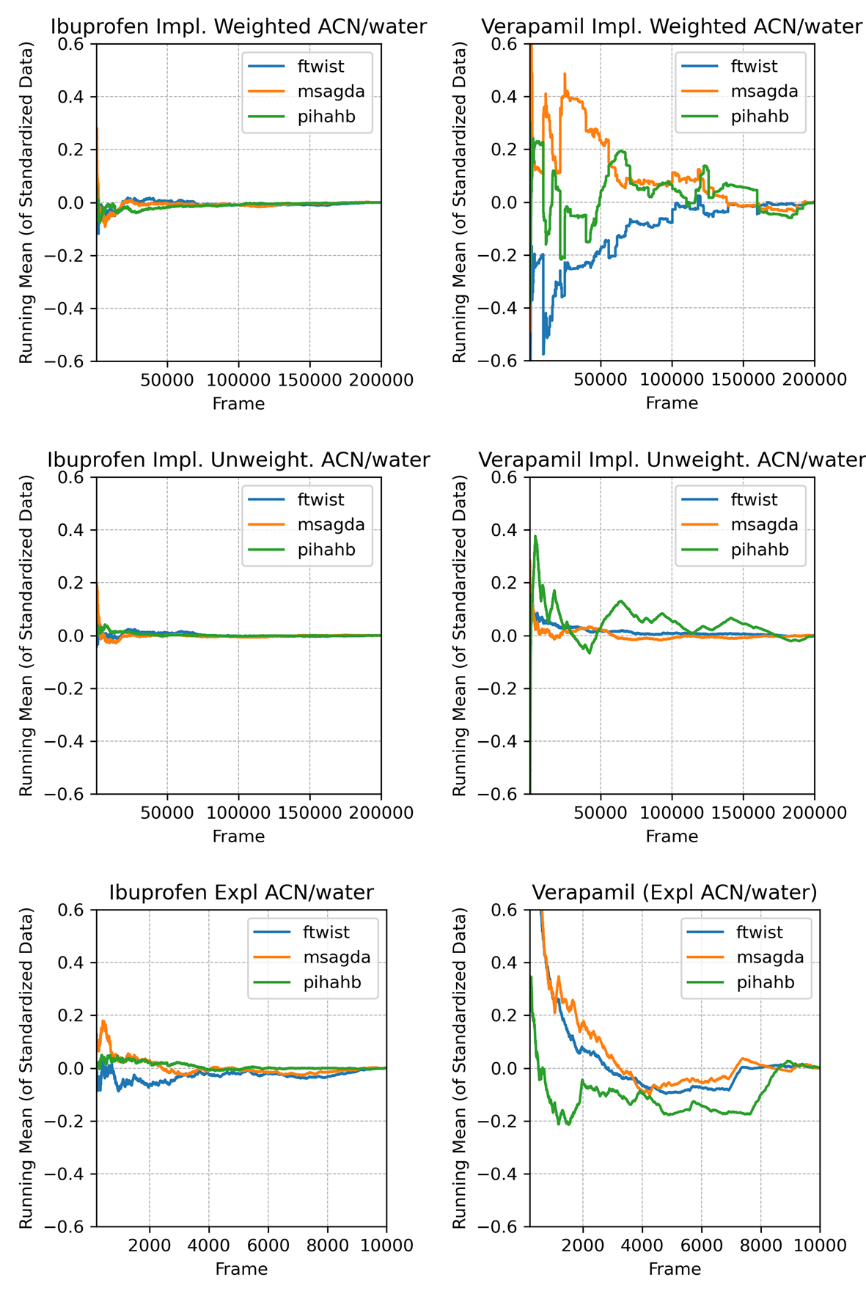


**S2 Fig.** **Running means of averaged descriptors *ftwist*, *msagda* and *pihahb* for ibuprofen (left) and verapamil (right).**

They are plotted as a function of the snapshot number (frequency 0.5 ps, top and middle rows; 10 ps bottom row). The top row shows running means of standardized conformation-dependent descriptor data (Z-scores) for implicit solvent simulation with SGLD weight factor applied. The middle row presents the same data without SGLD weight factors. The bottom row displays the results from the explicit solvent simulations.
