# Supplementary material for: Development of Ensemble Steric and Electrostatic Chirality (ESEC) descriptors for modelling chromatographic enantioseparations
Source: PLoS One. 2025 Oct 17;20(10):e0333635. doi: 10.1371/journal.pone.0333635 (PMC12533851; doi:10.1371/journal.pone.0333635)
Supplement: S3 Fig — (DOCX) [file pone.0333635.s005.docx]

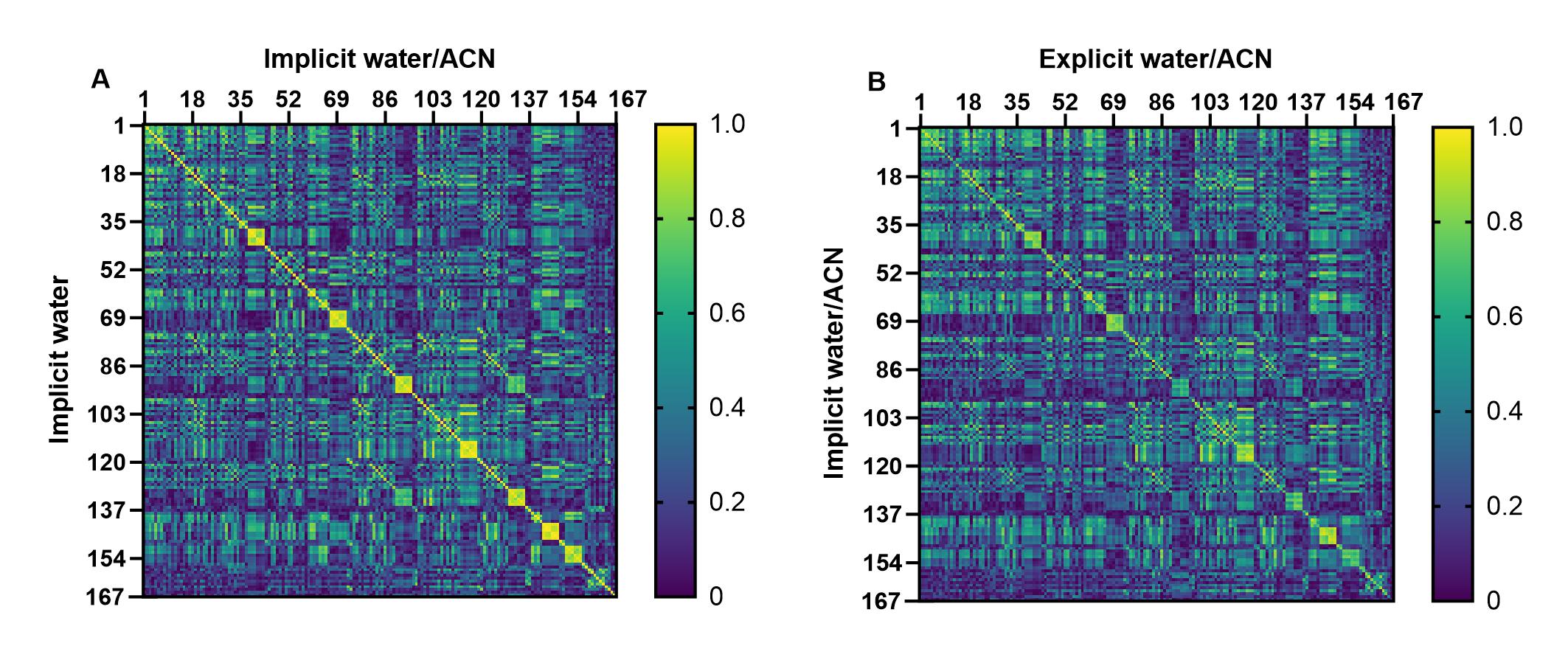


**S3 Fig**. **Heat map for the correlation coefficients calculated between the averaged chiral descriptors obtained from different MD simulations.**

(A) implicit water and water/ACN, and (B) implicit water/ACN and explicit water/ACN. Numbers 1 – 167 represent the number of each chiral descriptor, given in S5 Table.
