# Supplementary material for: Development of Ensemble Steric and Electrostatic Chirality (ESEC) descriptors for modelling chromatographic enantioseparations
Source: PLoS One. 2025 Oct 17;20(10):e0333635. doi: 10.1371/journal.pone.0333635 (PMC12533851; doi:10.1371/journal.pone.0333635)
Supplement: S5 Fig — (DOCX) [file pone.0333635.s007.docx]

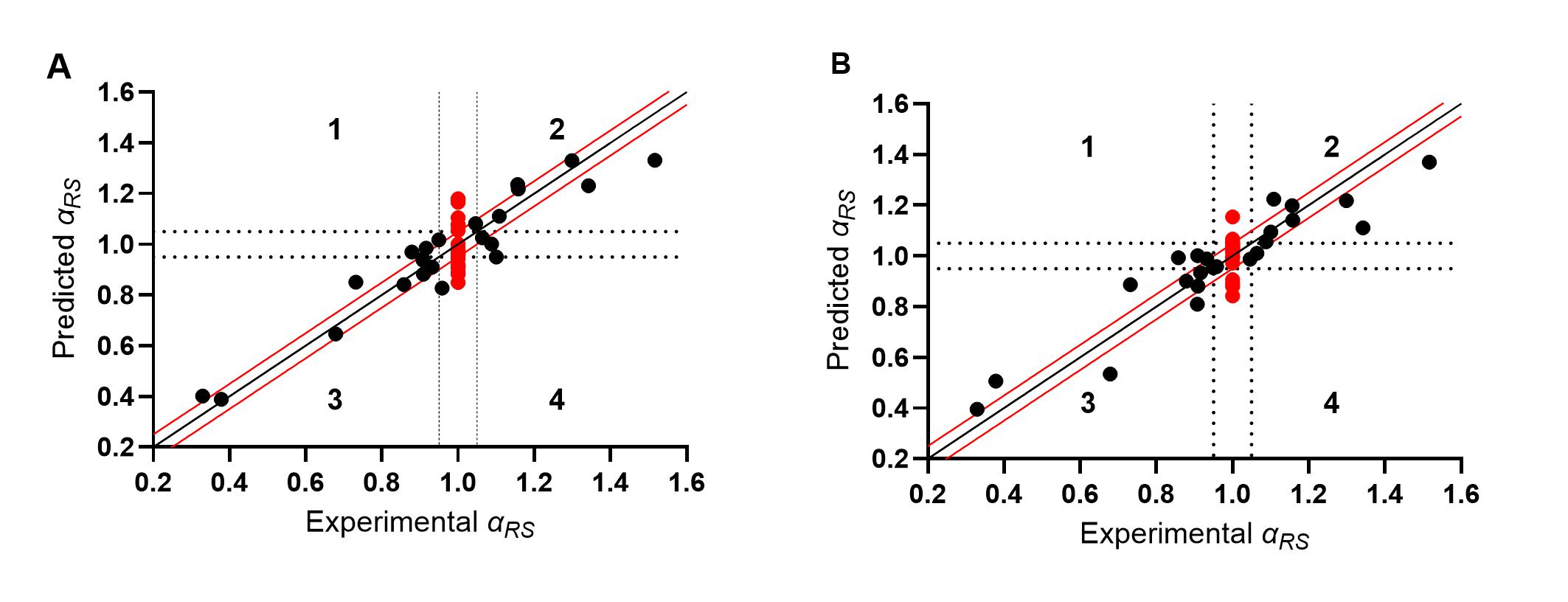


**S5 Fig.** **sMLR models: predicted *α_RS_* as a function of the experimental.**

Used descriptors: sets III, V, VI and VIII given in Table 2. Modelled responses: (A) log *α_RS_*, and (B) *α_RS_*. The dashed lines divide the graph into four quadrants (1, 2, 3 and 4) and the black full line is the bisector. The red lines are the limits for what is considered an accurate *α_RS_* prediction. Forty-two molecules were involved in the modelling and the red dots correspond to the experimentally unseparated molecules.
