# Supplementary material for: Development of Ensemble Steric and Electrostatic Chirality (ESEC) descriptors for modelling chromatographic enantioseparations
Source: PLoS One. 2025 Oct 17;20(10):e0333635. doi: 10.1371/journal.pone.0333635 (PMC12533851; doi:10.1371/journal.pone.0333635)
Supplement: S9 Fig — (DOCX) [file pone.0333635.s011.docx]

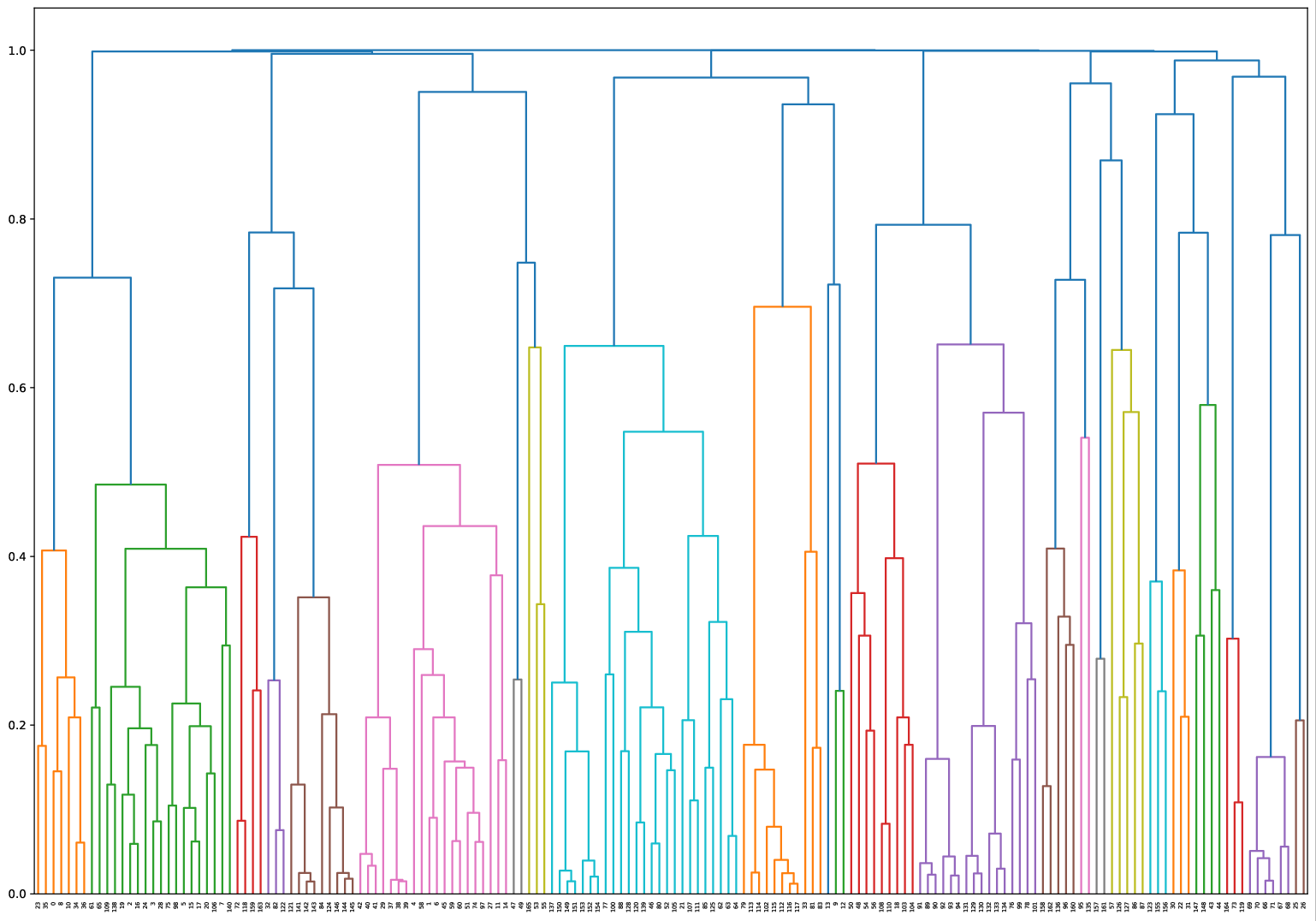


1-|r|

**S9 Fig.** **Dendrogram from the correlation coefficients calculated between the averaged chiral descriptors obtained from explicit water/ACN simulations.** Numbers 1 – 167: number of the chiral descriptors, given in S5 Table.
