# Supplementary material for: Development of Ensemble Steric and Electrostatic Chirality (ESEC) descriptors for modelling chromatographic enantioseparations
Source: PLoS One. 2025 Oct 17;20(10):e0333635. doi: 10.1371/journal.pone.0333635 (PMC12533851; doi:10.1371/journal.pone.0333635)
Supplement: S10 Fig — (DOCX) [file pone.0333635.s012.docx]

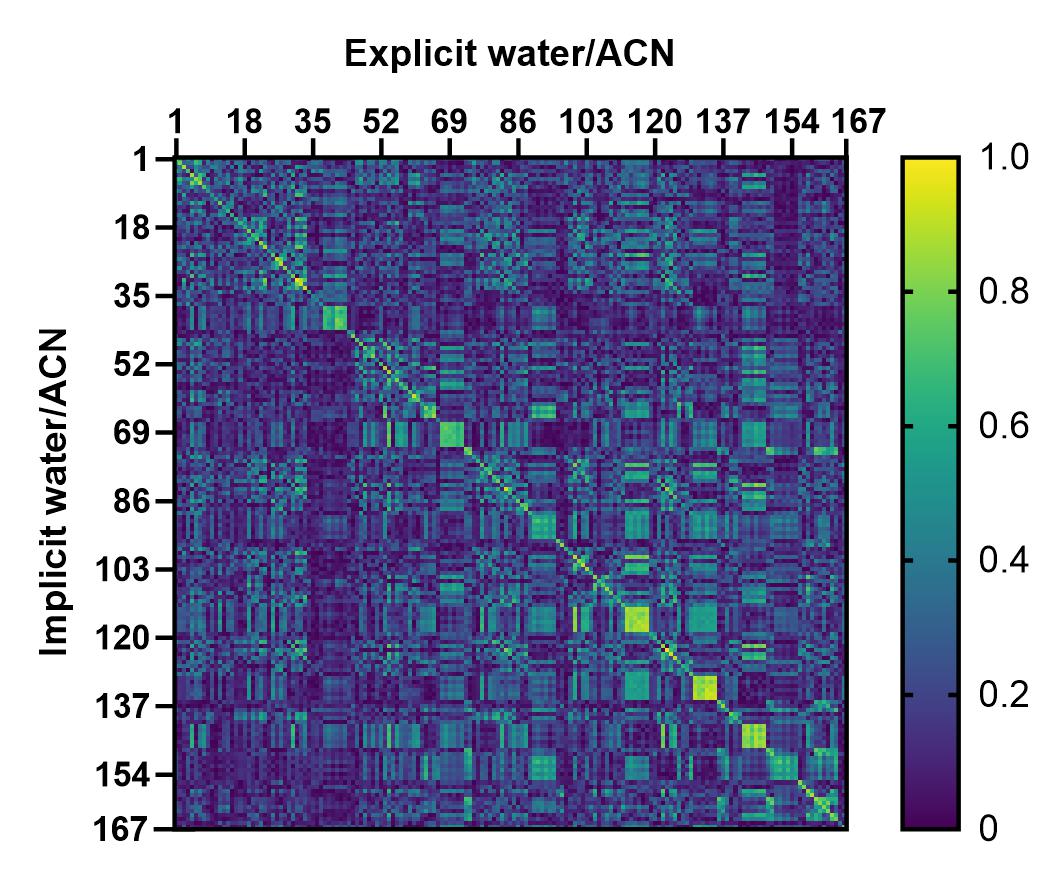


**S10 Fig**. **Heat map for the correlation coefficients calculated between the averaged chiral descriptors obtained from different MD simulations from the test set molecules in their uncharged state.**

Correlation between implicit water/ACN and explicit water/ACN. Numbers 1 – 167: number of a chiral descriptor, given in S5 Table.
