# Supplementary material for: Development of Ensemble Steric and Electrostatic Chirality (ESEC) descriptors for modelling chromatographic enantioseparations
Source: PLoS One. 2025 Oct 17;20(10):e0333635. doi: 10.1371/journal.pone.0333635 (PMC12533851; doi:10.1371/journal.pone.0333635)
Supplement: S1 Table — (DOCX) [file pone.0333635.s015.docx]

**S1 Table.** **Averaged chiral descriptors for R-medetomidine, protonated at pH 9 and simulated in implicit water/ACN.**

| Descriptor | Descriptor value | Descriptor | Descriptor value | Descriptor | Descriptor value | Descriptor | Descriptor value | Descriptor | Descriptor value |
| --- | --- | --- | --- | --- | --- | --- | --- | --- | --- |
| *mschac* | 0.635 ± 0.0016 | *mspihd* | -0.983 ± 0.0023 | *chhdha* | -0.0397 ± 0.00091 | *gsaghd* | 0.0515 ± 0.00022 | *aghada* | 0.0562 ± 0.00069 |
| *mschag* | 0.323 ± 0.0010 | ***mspiha*** | -1.18 ± 0.00076 | ***chhdhb*** | -0.0424 ± 0.00067 | ***gsagha*** | -0.0255 ± 0.00052 | ***aghbda*** | 0.100 ± 0.0010 |
| *mschal* | -0.609 ± 0.0016 | ***mspihb*** | -1.53 ± 0.0014 | ***chhdda*** | -0.0555 ± 0.0010 | ***gsaghb*** | 0.00952 ± 0.00033 | ***agsiso*** | -0.120 ± 0.0027 |
| *mschpi* | -0.0580 ± 0.0018 | ***mspida*** | -0.293 ± 0.0030 | ***chhahb*** | 0.00906 ± 0.00056 | ***gsagda*** | -0.0843 ± 0.00074 | ***agfifo*** | -0.0606 ± 0.0024 |
| *mschhd* | 0.435 ± 0.0014 | ***mshdha*** | 0.440 ± 0.0022 | ***chhada*** | -0.00503 ± 0.00089 | ***gsalpi*** | 0.314 ± 0.00089 | ***alpihd*** | -0.400 ± 0.0036 |
| *mschha* | 0.499 ± 0.0013 | ***mshdhb*** | 0.322 ± 0.0016 | ***chhbda*** | -0.0334 ± 0.0013 | ***gsalhd*** | -0.184 ± 0.00098 | ***alpiha*** | -0.133 ± 0.00098 |
| *mschhb* | 0.644 ± 0.0017 | ***mshdda*** | 0.503 ± 0.0025 | ***chsiso*** | 0.681 ± 0.0033 | ***gsalha*** | -0.0241 ± 0.00027 | ***alpihb*** | -0.275 ± 0.0016 |
| *mschda* | 0.124 ± 0.00079 | ***mshahb*** | -0.271 ± 0.0014 | ***chfifo*** | 0.918 ± 0.0035 | ***gsalhb*** | -0.122 ± 0.00044 | ***alpida*** | 0.122 ± 0.0044 |
| *msacgs* | -0.332 ± 0.00052 | ***mshada*** | 0.425 ± 0.0021 | ***acgsal*** | -0.0789 ± 0.00019 | ***gsalda*** | 0.139 ± 0.0012 | ***alhdha*** | 0.0343 ± 0.00042 |
| *msacal* | -0.406 ± 0.00063 | ***mshbda*** | 0.617 ± 0.0031 | ***acgspi*** | 0.303 ± 0.0010 | ***gspihd*** | 0.166 ± 0.0013 | ***alhdhb*** | 0.0198 ± 0.00031 |
| *msacpi* | 1.47 ± 0.00094 | ***mssiso*** | -0.0761 ± 0.0053 | ***acgshd*** | -0.148 ± 0.0010 | ***gspiha*** | 0.248 ± 0.00063 | ***alhdda*** | 0.0346 ± 0.00048 |
| *msachd* | -0.262 ± 0.0019 | ***msfifo*** | -0.0391 ± 0.0052 | ***acgsha*** | 0.0409 ± 0.00035 | ***gspihb*** | 0.301 ± 0.0012 | ***alhahb*** | -0.0271 ± 0.00027 |
| *msacha* | 0.339 ± 0.0010 | ***chacal*** | -0.0511 ± 0.00046 | ***acgshb*** | -0.0482 ± 0.00037 | ***gspida*** | 0.0613 ± 0.00082 | ***alhada*** | 0.0456 ± 0.00042 |
| *msachb* | 0.0945 ± 0.00046 | ***chacpi*** | 0.131 ± 0.0026 | ***acgsda*** | 0.169 ± 0.0013 | ***gshdha*** | -0.136 ± 0.0010 | ***alhbda*** | 0.0558 ± 0.00060 |
| *msacda* | 0.625 ± 0.0029 | ***chachd*** | 0.148 ± 0.0011 | ***acalpi*** | -0.00840 ± 0.00091 | ***gshdhb*** | -0.108 ± 0.00075 | ***alsiso*** | 0.210 ± 0.0029 |
| *msgsag* | 0.161 ± 0.00039 | ***chacha*** | 0.0914 ± 0.00015 | ***acalhd*** | -0.125 ± 0.0083 | ***gshdda*** | -0.161 ± 0.0012 | ***alfifo*** | 0.233 ± 0.0027 |
| *msgsal* | -0.303 ± 0.00049 | ***chachb*** | 0.140 ± 0.00072 | ***acalha*** | -0.0276 ± 0.00028 | ***gshahb*** | 0.0742 ± 0.00063 | ***pihdha*** | 0.242 ± 0.0038 |
| *msgspi* | -0.0892 ± 0.00056 | ***chacda*** | -0.00540 ± 0.0010 | ***acalhb*** | -0.0816 ± 0.00033 | ***gshada*** | -0.111 ± 0.0010 | ***pihdhb*** | 0.233 ± 0.0028 |
| *msgshd* | 0.236 ± 0.00064 | ***chagal*** | 0.103 ± 0.00077 | ***acalda*** | 0.0672 ± 0.0011 | ***gshbda*** | -0.177 ± 0.0014 | ***pihdda*** | 0.318 ± 0.0043 |
| *msgsha* | 0.248 ± 0.00038 | ***chagpi*** | 0.0774 ± 0.0016 | ***acpihd*** | 0.414 ± 0.0029 | ***gssiso*** | 0.367 ± 0.0025 | ***pihahb*** | -0.0860 ± 0.0023 |
| *msgshb* | 0.334 ± 0.00060 | ***chaghd*** | -0.0431 ± 0.00023 | ***acpiha*** | 0.119 ± 0.0016 | ***gsfifo*** | 0.443 ± 0.0025 | ***pihada*** | 0.0960 ± 0.0037 |
| *msgsda* | 0.0390 ± 0.00057 | ***chagha*** | -0.0531 ± 0.00078 | ***acpihb*** | 0.272 ± 0.00062 | ***agalpi*** | -0.238 ± 0.0012 | ***pihbda*** | 0.248 ± 0.0053 |
| *msagal* | 0.0131 ± 0.00083 | ***chaghb*** | -0.0758 ± 0.00067 | ***acpida*** | -0.181 ± 0.0046 | ***agalhd*** | 0.120 ± 0.00070 | ***pisiso*** | -1.40 ± 0.0052 |
| *msagpi* | 0.801 ± 0.00099 | ***chagda*** | -0.0290 ± 0.00082 | ***achdha*** | -0.119 ± 0.00075 | ***agalha*** | -0.00351 ± 0.00023 | ***pififo*** | -1.62 ± 0.0050 |
| *msaghd* | -0.282 ± 0.00072 | ***chalpi*** | -0.0981 ± 0.0026 | ***achdhb*** | -0.0941 ± 0.00055 | ***agalhb*** | 0.0639 ± 0.00032 | ***hdsiso*** | 0.478 ± 0.0039 |
| *msagha* | -0.00849 ± 0.00095 | ***chalhd*** | -0.0858 ± 0.00079 | ***achdda*** | -0.141 ± 0.00086 | ***agalda*** | -0.102 ± 0.00086 | ***hdfifo*** | 0.757 ± 0.0038 |
| *msaghb* | -0.190 ± 0.00036 | ***chalha*** | -0.0557 ± 0.00042 | ***achahb*** | 0.0646 ± 0.00046 | ***agpihd*** | 0.0395 ± 0.0012 | ***hasiso*** | -0.251 ± 0.0031 |
| *msagda* | 0.278 ± 0.0017 | ***chalhb*** | -0.0766 ± 0.00039 | ***achada*** | -0.0963 ± 0.00071 | ***agpiha*** | -0.115 ± 0.0016 | ***hafifo*** | -0.317 ± 0.0030 |
| *msalpi* | -1.46 ± 0.00086 | ***chalda*** | 0.00137 ± 0.0011 | ***achbda*** | -0.154 ± 0.0010 | ***agpihb*** | -0.105 ± 0.00047 | ***hbsiso*** | 0.103 ± 0.0032 |
| *msalhd* | 0.527 ± 0.0023 | ***chpihd*** | -0.161 ± 0.0021 | ***acsiso*** | 0.149 ± 0.0018 | ***agpida*** | -0.142 ± 0.0030 | ***hbfifo*** | 0.220 ± 0.0028 |
| *msalha* | -0.00553 ± 0.00056 | ***chpiha*** | -0.0694 ± 0.0023 | ***acfifo*** | 0.231 ± 0.0016 | ***aghdha*** | 0.0824 ± 0.00073 | ***dasiso*** | -0.716 ± 0.0051 |
| *msalhb* | 0.325 ± 0.0010 | ***chpihb*** | -0.149 ± 0.0029 | ***gsagal*** | 0.0484 ± 0.00048 | ***aghdhb*** | 0.0695 ± 0.00054 | ***dafifo*** | -1.05 ± 0.0054 |
| *msalda* | -0.523 ± 0.0028 | ***chpida*** | -0.0221 ± 0.00056 | ***gsagpi*** | -0.155 ± 0.00075 | ***aghdda*** | 0.101 ± 0.00083 | ***stwist*** | -0.249 ± 0.0055 |
|  |  |  |  |  |  | ***aghahb*** | -0.0402 ± 0.00044 | ***ftwist*** | -0.547 ± 0.0070 |
| SEM = standard error on the mean. | | | |  |  |  |  |  |  |

Values are expressed as mean ± SEM (based on 200,000 values).
