# Supplementary material for: Development of Ensemble Steric and Electrostatic Chirality (ESEC) descriptors for modelling chromatographic enantioseparations
Source: PLoS One. 2025 Oct 17;20(10):e0333635. doi: 10.1371/journal.pone.0333635 (PMC12533851; doi:10.1371/journal.pone.0333635)
Supplement: S2 Table — (DOCX) [file pone.0333635.s016.docx]

**S2 Table.** **Chromatographic results with 0.02 M borate buffer pH 9 / ACN (60/40 V/V) on Lux amylose-2.**

| Compound | Enantiomer | *t_R_* (min) | *k* | *α_RS_* | Log *α_RS_* |
| --- | --- | --- | --- | --- | --- |
| Acenocoumarol | R | 6.59 | 0.186 | 1.00 | 0.00 |
|  | S | 6.59 | 0.186 |  |  |
| Aminogluthetimide | R | 23.5 | 3.22 | 1.34 | 0.13 |
|  | S | 18.9 | 2.40 |  |  |
| Atenolol | R | 8.58 | 0.543 | 1.00 | 0.00 |
|  | S | 8.58 | 0.543 |  |  |
| Baclofen | R | 5.68 | 0.0216 | 1.00 | 0.00 |
|  | S | 5.68 | 0.0216 |  |  |
| Blebbistatine | R | 63.5 | 10.4 | 1.09 | 0.037 |
|  | S | 58.8 | 9.58 |  |  |
| Bupivacaine | R | 69.8 | 11.5 | 1.00 | 0.00 |
|  | S | 69.8 | 11.5 |  |  |
| Carbinoxamine | R | 23.5 | 3.23 | 0.916 | -0.038 |
|  | S | 25.2 | 3.53 |  |  |
| Carvone | R | 24.7 | 3.44 | 0.958 | -0.019 |
|  | S | 25.5 | 3.59 |  |  |
| Cetirizine | R | 114 | 19.5 | 1.16 | 0.063 |
|  | S | 99.3 | 16.9 |  |  |
| Clopidogrel | R | 116 | 19.9 | 1.00 | 0.00 |
|  | S | 116 | 19.9 |  |  |
| Equol | R | 10.4 | 0.873 | 1.00 | 0.00 |
|  | S | 10.4 | 0.873 |  |  |
| Etiracetam | R | 6.55 | 0.179 | 1.00 | 0.00 |
|  | S | 6.55 | 0.179 |  |  |
| Fluoxetine | R | 63.9 | 10.5 | 1.06 | 0.027 |
|  | S | 60.4 | 9.87 |  |  |
| Ibuprofen | R | 5.75 | 0.0336 | 1.00 | 0.00 |
|  | S | 5.75 | 0.0336 |  |  |
| Indapamide | R | 13.2 | 1.37 | 1.16 | 0.064 |
|  | S | 12.1 | 1.18 |  |  |
| Isoxanthohumol | R | 5.75 | 0.0336 | 1.00 | 0.00 |
|  | S | 5.75 | 0.0336 |  |  |
| Ketoprofen | R | 5.70 | 0.0252 | 1.00 | 0.00 |
|  | S | 5.70 | 0.0252 |  |  |
| Lansoprazole | R | 6.67 | 0.200 | 0.679 | -0.17 |
|  | S | 7.20 | 0.295 |  |  |
| Laudanosine | R | 21.7 | 2.90 | 1.30 | 0.11 |
|  | S | 18.0 | 2.23 |  |  |
| Lisofylline | R | 7.42 | 0.335 | 1.00 | 0.00 |
|  | S | 7.42 | 0.335 |  |  |
| Mandelic acid | **R** | **5.56** | **0** | **/** | / |
|  | **S** | **5.56** | **0** |  |  |
| Medetomidine | R | 14.9 | 1.68 | 0.907 | -0.042 |
|  | S | 15.9 | 1.85 |  |  |
| Metalaxyl | R | 17.5 | 2.14 | 1.11 | 0.044 |
|  | S | 16.3 | 1.94 |  |  |
| Modafinil | R | 10.2 | 0.830 | 0.909 | -0.041 |
|  | S | 10.6 | 0.912 |  |  |
| Ofloxacine | R | 5.63 | 0.0131 | 1.00 | 0.00 |
|  | S | 5.63 | 0.0131 |  |  |
| Omeprazole | R | 7.00 | 0.259 | 1.00 | 0.00 |
|  | S | 7.00 | 0.259 |  |  |
| Ondansetron | R | 18.6 | 2.35 | 1.00 | 0.00 |
|  | S | 18.6 | 2.35 |  |  |
| Piperitone | R | 21.4 | 2.84 | 0.950 | -0.022 |
|  | S | 22.2 | 2.99 |  |  |
| Pramipexole | R | 10.5 | 0.884 | 0.330 | -0.48 |
|  | S | 20.5 | 2.68 |  |  |
| Praziquantel | R | 68.0 | 11.2 | 1.52 | 0.18 |
|  | S | 46.7 | 7.40 |  |  |
| Propranolol | R | 22.2 | 2.98 | 0.858 | -0.067 |
|  | S | 24.9 | 3.48 |  |  |
| Razoxane | R | 6.41 | 0.152 | 1.00 | 0.00 |
|  | S | 6.41 | 0.152 |  |  |
| Rivaroxaban | R | 18.3 | 2.28 | 0.731 | -0.14 |
|  | S | 22.9 | 3.12 |  |  |
| Rolipram | R | 14.5 | 1.61 | 1.10 | 0.042 |
|  | S | 15.4 | 1.77 |  |  |
| Salbutamol | R | 6.39 | 0.150 | 1.00 | 0.00 |
|  | S | 6.39 | 0.150 |  |  |
| Sulpiride | R | 9.40 | 0.691 | 1.00 | 0.00 |
|  | S | 9.40 | 0.691 |  |  |
| Tamsulosine | R | 19.9 | 2.59 | 0.909 | -0.042 |
|  | S | 21.4 | 2.85 |  |  |
| Tetramisole | R | 24.6 | 3.43 | 0.879 | -0.056 |
|  | S | 27.2 | 3.90 |  |  |
| Thalidomide | R | 13.6 | 1.44 | 0.933 | -0.030 |
|  | S | 14.2 | 1.55 |  |  |
| Tipifarnib | R | 136 | 23.40 | 1.00 | 0.00 |
|  | S | 136 | 23.40 |  |  |
| Tolterodine | R | 52.5 | 8.44 | 1.00 | 0.00 |
|  | S | 52.5 | 8.44 |  |  |
| Verapamil | R | 60.1 | 9.81 | 1.05 | 0.019 |
|  | S | 57.7 | 9.38 |  |  |
| Warfarine | R | 5.87 | 0.0563 | 0.378 | -0.42 |
|  | S | 6.39 | 0.149 |  |  |

*t_0_* = 5.56 min.
