# Supplementary material for: Development of Ensemble Steric and Electrostatic Chirality (ESEC) descriptors for modelling chromatographic enantioseparations
Source: PLoS One. 2025 Oct 17;20(10):e0333635. doi: 10.1371/journal.pone.0333635 (PMC12533851; doi:10.1371/journal.pone.0333635)
Supplement: S3 Table — (DOCX) [file pone.0333635.s017.docx]

**S3 Table.** **Overview of sMLR models for enantioselectivity, built with different types and combinations of chiral descriptors.**

| (A) Chiral descriptors calculated from MD simulations in **implicit** solvent | | | | | | | | | | |
| --- | --- | --- | --- | --- | --- | --- | --- | --- | --- | --- |
| **Averaged charged descriptors (weighted averages)** | | | | | | | | | | |
| **Solvent**  **system** | **Descriptors** | | **RMSECV_N_** | **RMSEC_N_** | **r^2^** | **q^2^** | **Prediction error (%)** | **Accurate predictions** | **Correct predictions** | **Elution sequence** |
| Water | 4 | | 0.181 | 0.152 | 0.1988 | -0.2737 | 17.48 | 10/42 | 24/42 | 15/23 |
|  | **Equation** | | Log *α_RS_* = -0.020 - 0.0041 *achdda* + 0.037 *achada* + 0.021 *chalpi* - 0.028 *msagda* (S3) | | | | | | | |
| Water/  ACN | 4 | | 0.172 | 0.144 | 0.2764 | -0.2308 | 16.75 | 11/42 | 23/42 | 16/23 |
|  | **Equation** | | Log *α_RS_* = -0.020 + 0.032 *chalpi* + 0.041 *achdda* - 0.030 *ftwist* - 0.012 *msagda* (S4) | | | | | | | |
| **Averaged charged descriptors (unweighted averages)** | | | | | | | | | | |
| **Solvent system** | **Descriptors** | | **RMSECV_N_** | **RMSEC_N_** | **r^2^** | **q^2^** | **Prediction error (%)** | **Accurate predictions** | **Correct predictions** | **Elution sequence** |
| Water | 4 | | 0.179 | 0.144 | 0.2849 | -0.4719 | 16.38 | 11/42 | 23/42 | 14/23 |
|  | **Equation** | | Log *α_RS_* = -0.020 + 0.052 *achdda* + 0.031 *chpida* + 0.013 *chalpi* + 0.022 *alpihd* (S5) | | | | | | | |
| Water/  ACN | 4 | | 0.189 | 0.139 | 0.3311 | -0.4023 | 16.20 | 12/42 | 22/42 | 15/23 |
|  | **Equation** | | Log *α_RS_ =* -0.020 *+* 0.037 *chalpi +* 0.062 *achdda +* 0.043 *gsalhb -* 0.013 *alpida* (S6) | | | | | | | |
| (B) Chiral descriptors calculated from MD simulations in **explicit** solvent | | | | | | | | | | |
| **Averaged charged descriptors** | | | | | | | | | | |
| **Solvent system** | **Descriptors** | | **RMSECV_N_** | **RMSEC_N_** | **r^2^** | **q^2^** | **Prediction error (%)** | **Accurate predictions** | **Correct predictions** | **Elution sequence** |
| Water/  ACN | 4 | | 0.135 | 0.118 | 0.5169 | 0.2534 | 12.47 | 17/42 | 25/42 | 14/23 |
|  | **Equation** | | Log *α_RS_* = -0.020 + 0.069 *achbda* - 0.088 *chpihd* - 0.099 *gsagal* - 0.048 *msagda* (S7) | | | | | | | |
| **Windowed charged descriptors** | | | | | | | | | | |
| **Solvent system** | **Descriptors** | | **RMSECV_N_** | **RMSEC_N_** | **r^2^** | **q^2^** | **Prediction error (%)** | **Accurate predictions** | **Correct predictions** | **Elution sequence** |
| Water/  ACN | 5 | | 0.137 | 0.114 | 0.5488 | 0.2165 | 13.47 | 11/42 | 21/42 | 16/23 |
|  | **Equation** | | Log *α_RS_* = -0.020 - 0.087 *msaghd-* + 0.073 *acsiso+* + 0.052 *acalhb+* + 0.059 *chagal-*  + 0.046 *msachd+* (S8) | | | | | | | |
| (C) Chiral descriptors calculated from MD simulations in **implicit and** **explicit** solvent | | | | | | | | | | |
| **Averaged charged descriptors (weighted averages)** | | | | | | | | | | |
| **Solvent system** | **Descriptors** | | **RMSECV_N_** | **RMSEC_N_** | **r^2^** | **q^2^** | **Prediction error (%)** | **Accurate predictions** | **Correct predictions** | **Elution sequence** |
| Water/  ACN | 5 | | 0.134 | 0.112 | 0.5689 | 0.1747 | 12.38 | 13/42 | 26/42 | 16/23 |
|  | **Equation** | | Log *α_RS_* = -0.020 + 0.078 *achbda (explicit)* - 0.092 *msagda (implicit water/ACN)* + 0.092 *mshdhb (implicit water)* + 0.055 *aghdda (implicit water)* + 0.039 *chachb (implicit water/ACN)* (S9) | | | | | | | |
|  | | **Windowed charged descriptors (weighted averages)** | | | | | | | | |
| **Solvent system** | **Descriptors** | | **RMSECV_N_** | **RMSEC_N_** | **r^2^** | **q^2^** | **Prediction error (%)** | **Accurate predictions** | **Correct predictions** | **Elution sequence** |
| Water/  ACN | 7 | | 0.104 | 0.0853 | 0.7475 | 0.4963 | 10.22 | 15/42 | 26/42 | 18/23 |
|  | **Equation** | | Log *α_RS_* = - 0.020 - 0.058 *msaghd- (explicit)* + 0.067 *acsiso+ (explicit)* + 0.058 *acalhb+ (explicit)* + 0.088 *chagal- (explicit)* + 0.050 *aghahb- (implicit water/ACN)* - 0.051 *ftwist+ (explicit)* - 0.042 *alhada- (explicit)* (S10) | | | | | | | |
| **Averaged charged descriptors (unweighted averages)** | | | | | | | | | | |
| **Solvent system** | **Descriptors** | | **RMSECV_N_** | **RMSEC_N_** | **r^2^** | **q^2^** | **Prediction error (%)** | **Accurate predictions** | **Correct predictions** | **Elution sequence** |
| Water/  ACN | 5 | | 0.159 | 0.113 | 0.5567 | -0.6588 | 13.16 | 12/42 | 20/42 | 17/23 |
|  | **Equation** | | Log *α_RS_* = - 0.020 + 0.062 *achbda (explicit water/ACN)* - 0.63 *msagda (implicit water/ACN)* + 0.61 *msagda (implicit water)* - 0.050 *chhdhb* (explicit water/ACN) - 0.046 *gsalpi (explicit water/ACN)* (S11) | | | | | | | |
| **Windowed charged descriptors (unweighted averages)** | | | | | | | | | | |
| **Solvent system** | **Descriptors** | | **RMSECV_N_** | **RMSEC_N_** | **r^2^** | **q^2^** | **Prediction error (%)** | **Accurate predictions** | **Correct predictions** | **Elution sequence** |
| Water/  ACN | 7 | | 0.124 | 0.0979 | 0.6679 | 0.3263 | 12.66 | 7/42 | 22/42 | 16/23 |
|  | **Equation** | | Log *α_RS_* = - 0.020 - 0.052 *msaghd- (explicit water/ACN)* + 0.051 *acsiso+ (explicit water/ACN)* + 0.043 *chagal- (explicit water/ACN)* + 0.047 *aghahb- (implicit water/ACN)* - 0.063 *ftwist+ (explicit water/ACN)* - 0.044 *alhada- (explicit water/ACN)* - 0.028 *agpida- (explicit water/ACN)* (S12) | | | | | | | |
| (D) Chiral descriptors calculated from MD simulations in **implicit and explicit** solvent | | | | | | | | | | |
| **Averaged uncharged descriptors (weighted averages)** | | | | | | | | | | |
| **Solvent system** | **Descriptors** | | **RMSECV_N_** | **RMSEC_N_** | **r^2^** | **q^2^** | **Prediction error (%)** | **Accurate predictions** | **Correct predictions** | **Elution sequence** |
| Water/  ACN | 6 | | 0.0992 | 0.0758 | 0.8009 | 0.3979 | 9.03 | 14/42 | 21/42 | 21/23 |
|  | **Equation** | | Log *α_RS_* = - 0.020 - 0.14 *msachd (explicit)* + 0.10 *msachd (implicit)* + 0.040 *mshada (implicit)* - 0.055 *msgsal (explicit)* - 0.062 *gspida (explicit)* - 0.035 *msaghb (implicit)*(S13) | | | | | | | |
| **Windowed uncharged descriptors (weighted averages)** | | | | | | | | | | |
| **Solvent system** | **Descriptors** | | **RMSECV_N_** | **RMSEC_N_** | **r^2^** | **q^2^** | **Prediction error (%)** | **Accurate predictions** | **Correct predictions** | **Elution sequence** |
| Water/  ACN | 9 | | 0.0708 | 0.0510 | 0.9098 | 0.7292 | 6.23 | 21/42 | 26/42 | 22/23 |
|  | **Equation** | | Log *α_RS_* = -0.020 - 0.046 *msgshb- (explicit)* + 0.042 *agsiso+ (explicit)* - 0.052 *msgsha+ (implicit)* + 0.066 *pisiso- (explicit)* - 0.040 *chagha- (explicit)* - 0.070 *msaghd+ (explicit)* + 0.022 *msalha- (implicit)* - 0.030 *acgsha+ (explicit)* + 0.031 *acgshd-* *(implicit)* (S14) | | | | | | | |
| **Averaged and windowed uncharged descriptors (weighted averages)** | | | | | | | | | | |
| **Solvent system** | **Descriptors** | | **RMSECV_N_** | **RMSEC_N_** | **r^2^** | **q^2^** | **Prediction error (%)** | **Accurate predictions** | **Correct predictions** | **Elution sequence** |
| Water/  ACN | 7 | | 0.0806 | 0.0603 | 0.8739 | 0.6782 | 7.11 | 19/42 | 23/42 | 20/23 |
|  | **Equation** | | Log *α_RS_* = -0.020 - 0.080 *msgshb- (explicit)* + 0.040 *agsiso+ (explicit)* - 0.068 *msgsha+ (implicit)* + 0.052 *pisiso- (explicit)* - 0.040 *chagha- (explicit)* - 0.031 *msaghd+ (explicit)* - 0.023 *gsalpi (explicit)* (S15) | | | | | | | |
| **Averaged and windowed uncharged descriptors (unweighted averages)** | | | | | | | | | | |
| **Solvent system** | **Descriptors** | | **RMSECV_N_** | **RMSEC_N_** | **r^2^** | **q^2^** | **Prediction error (%)** | **Accurate predictions** | **Correct predictions** | **Elution sequence** |
| Water/  ACN | 8 | | 0.109 | 0.0751 | 0.8043 | 0.4130 | 10.06 | 9/42 | 19/42 | 18/23 |
|  | **Equation** | | Log *α_RS_* = -0.020 - 0.061 *msgshb- (explicit)* + 0.038 *agsiso+ (explicit)* - 0.043 *acgspi+ (explicit)* + 0.047 *pisiso- (explicit)* - 0.019 *msacpi- (explicit)* + 0.030 *msaghb- (explicit)* + 0.027 *chagda+ (explicit)* - 0.019 *acalhb+ (explicit)* (S16) | | | | | | | |

n = 42, with 23 molecules experimentally separated.
