# Supplementary material for: Development of Ensemble Steric and Electrostatic Chirality (ESEC) descriptors for modelling chromatographic enantioseparations
Source: PLoS One. 2025 Oct 17;20(10):e0333635. doi: 10.1371/journal.pone.0333635 (PMC12533851; doi:10.1371/journal.pone.0333635)
Supplement: S4 Table — (DOCX) [file pone.0333635.s018.docx]

**S4 Table.** **Overview of PLS models for enantioselectivity, built with different types and combinations of chiral descriptors.**

| (A) Chiral descriptors calculated from MD simulations in **implicit** solvent | | | | | | | |
| --- | --- | --- | --- | --- | --- | --- | --- |
| **averaged charged descriptors (weighted averages)** | | | | | | | |
| **Solvent system** | **RMSECV_N_** | **RMSEC_N_** | **r^2^** | **Prediction error (%)** | **Accurate predictions** | **Correct predictions** | **Elution sequence** |
| Water | 0.197 | 0.155 | 0.1690 | 16.50 | 14/42 | 22/42 | 17/23 |
| Water  /ACN | 0.190 | 0.156 | 0.1555 | 16.71 | 12/42 | 19/42 | 16/23 |
| **averaged charged descriptors (unweighted averages)** | | | | | | | |
| **Solvent system** | **RMSECV_N_** | **RMSEC_N_** | **r^2^** | **Prediction error (%)** | **Accurate predictions** | **Correct predictions** | **Elution sequence** |
| Water | 0.206 | 0.144 | 0.2786 | 16.16 | 12/42 | 20/42 | 18/23 |
| Water  /ACN | 0.203 | 0.143 | 0.2874 | 16.01 | 14/42 | 22/42 | 18/23 |
| (B) Chiral descriptors calculated from MD simulations in **explicit** solvent | | | | | | | |
| **averaged charged descriptors** | | | | | | | |
| **Solvent system** | **RMSECV_N_** | **RMSEC_N_** | **r^2^** | **Prediction error (%)** | **Accurate predictions** | **Correct predictions** | **Elution sequence** |
| Water  /ACN | 0.178 | 0.156 | 0.1523 | 15.98 | 22/42 | 27/42 | 17/23 |
| **Windowed charged descriptors** | | | | | | | |
| **Solvent system** | **RMSECV_N_** | **RMSEC_N_** | **r^2^** | **Prediction error (%)** | **Accurate predictions** | **Correct predictions** | **Elution sequence** |
| Water  /ACN | 0.179 | 0.139 | 0.3326 | 16.35 | 9/42 | 24/42 | 17/23 |

n = 42, with 23 molecules experimentally separated. For all models, 1 PLS factor was selected.
