# Supplementary material for: Development of Ensemble Steric and Electrostatic Chirality (ESEC) descriptors for modelling chromatographic enantioseparations
Source: PLoS One. 2025 Oct 17;20(10):e0333635. doi: 10.1371/journal.pone.0333635 (PMC12533851; doi:10.1371/journal.pone.0333635)
Supplement: S5 Table — (DOCX) [file pone.0333635.s019.docx]

**S5 Table.** **Number related to a given averaged chiral descriptor.**

| Number | Descriptor | Number | Descriptor | Number | Descriptor | Number | Descriptor | Number | Descriptor |
| --- | --- | --- | --- | --- | --- | --- | --- | --- | --- |
| 1 | *mschac* | **35** | *mspiha* | **69** | *chhdda* | **102** | *gsaghb* | **135** | *aghbda* |
| 2 | *mschag* | **36** | *mspihb* | **70** | *chhahb* | **103** | *gsagda* | **136** | *agsiso* |
| 3 | *mschal* | **37** | *mspida* | **71** | *chhada* | **104** | *gsalpi* | **137** | *agfifo* |
| 4 | *mschpi* | **38** | *mshdha* | **72** | *chhbda* | **105** | *gsalhd* | **138** | *alpihd* |
| 5 | *mschhd* | **39** | *mshdhb* | **73** | *chsiso* | **106** | *gsalha* | **139** | *alpiha* |
| 6 | *mschha* | **40** | *mshdda* | **74** | *chfifo* | **107** | *gsalhb* | **140** | *alpihb* |
| 7 | *mschhb* | **41** | *mshahb* | **75** | *acgsal* | **108** | *gsalda* | **141** | *alpida* |
| 8 | *mschda* | **42** | *mshada* | **76** | *acgspi* | **109** | *gspihd* | **142** | *alhdha* |
| 9 | *msacgs* | **43** | *mshbda* | **77** | *acgshd* | **110** | *gspiha* | **143** | *alhdhb* |
| 10 | *msacal* | **44** | *mssiso* | **78** | *acgsha* | **111** | *gspihb* | **144** | *alhdda* |
| 11 | *msacpi* | **45** | *msfifo* | **79** | *acgshb* | **112** | *gspida* | **145** | *alhahb* |
| 12 | *msachd* | **46** | *chacal* | **80** | *acgsda* | **113** | *gshdha* | **146** | *alhada* |
| 13 | *msacha* | **47** | *chacpi* | **81** | *acalpi* | **114** | *gshdhb* | **147** | *alhbda* |
| 14 | *msachb* | **48** | *chachd* | **82** | *acalhd* | **115** | *gshdda* | **148** | *alsiso* |
| 15 | *msacda* | **49** | *chacha* | **83** | *acalha* | **116** | *gshahb* | **149** | *alfifo* |
| 16 | *msgsag* | **50** | *chachb* | **84** | *acalhb* | **117** | *gshada* | **150** | *pihdha* |
| 17 | *msgsal* | **51** | *chacda* | **85** | *acalda* | **118** | *gshbda* | **151** | *pihdhb* |
| 18 | *msgspi* | **52** | *chagal* | **86** | *acpihd* | **119** | *gssiso* | **152** | *pihdda* |
| 19 | *msgshd* | **53** | *chagpi* | **87** | *acpiha* | **120** | *gsfifo* | **153** | *pihahb* |
| 20 | *msgsha* | **54** | *chaghd* | **88** | *acpihb* | **121** | *agalpi* | **154** | *pihada* |
| 21 | *msgshb* | **55** | *chagha* | **89** | *acpida* | **122** | *agalhd* | **155** | *pihbda* |
| 22 | *msgsda* | **56** | *chaghb* | **90** | *achdha* | **123** | *agalha* | **156** | *pisiso* |
| 23 | *msagal* | **57** | *chagda* | **91** | *achdhb* | **124** | *agalhb* | **157** | *pififo* |
| 24 | *msagpi* | **58** | *chalpi* | **92** | *achdda* | **125** | *agalda* | **158** | *hdsiso* |
| 25 | *msaghd* | **59** | *chalhd* | **93** | *achahb* | **126** | *agpihd* | **159** | *hdfifo* |
| 26 | *msagha* | **60** | *chalha* | **94** | *achada* | **127** | *agpiha* | **160** | *hasiso* |
| 27 | *msaghb* | **61** | *chalhb* | **95** | *achbda* | **128** | *agpihb* | **161** | *hafifo* |
| 28 | *msagda* | **62** | *chalda* | **96** | *acsiso* | **129** | *agpida* | **162** | *hbsiso* |
| 29 | *msalpi* | **63** | *chpihd* | **97** | *acfifo* | **130** | *aghdha* | **163** | *hbfifo* |
| 30 | *msalhd* | **64** | *chpiha* | **98** | *gsagal* | **131** | *aghdhb* | **164** | *dasiso* |
| 31 | *msalha* | **65** | *chpihb* | **99** | *gsagpi* | **132** | *aghdda* | **165** | *dafifo* |
| 32 | *msalhb* | **66** | *chpida* | **100** | *gsaghd* | **133** | *aghahb* | **166** | *stwist* |
| 33 | *msalda* | **67** | *chhdha* | **101** | *gsagha* | **134** | *aghada* | **167** | *ftwist* |
| 34 | *mspihd* | **68** | *chhdhb* |  | | | | | |

The same numbers are applied for the negatively and positively windowed descriptors.
