# Supplementary material for: Development of Ensemble Steric and Electrostatic Chirality (ESEC) descriptors for modelling chromatographic enantioseparations
Source: PLoS One. 2025 Oct 17;20(10):e0333635. doi: 10.1371/journal.pone.0333635 (PMC12533851; doi:10.1371/journal.pone.0333635)
Supplement: S6 Table — (DOCX) [file pone.0333635.s020.docx]

**S6 Table.** **sMLR models built with different types and combinations of unweighted chiral descriptors (sets III, V, VI and VIII).**

| **Solvent system** | **Descriptors** | **RMSECV_N_** | **RMSEC_N_** | **r^2^** | **q^2^** | **Prediction error (%)** | **Accurate predictions** | **Correct predictions** | **Elution sequence** |
| --- | --- | --- | --- | --- | --- | --- | --- | --- | --- |
| Water  /ACN | 7 | 0.0797 | 0.0609 | 0.8712 | 0.6359 | 7.82 | 16/42 | 21/42 | 21/23 |
|  | **Equation** | Log *α_RS_* = -0.020 - 0.047 msgshb*- (explicit uncharged)* + 0.075 *agsiso+ (explicit uncharged)* + 0.051 *achbda (implicit charged)* - 0.071 *gsalhd- (explicit uncharged)* - 0.029 *msagal- (explicit charged)* + 0.035 *aghdhb- (explicit uncharged)* - 0.035 *hasiso (implicit uncharged)* (S17) | | | | | | | |
| Water  /ACN | 7 | 0.0972 | 0.0730 | 0.8067 | 0.7036 | 7.63 | 20/42 | 29/42 | 21/23 |
|  | Equation | *α_RS_* = 0.98 - 0.13 *msgshb- (explicit uncharged)* + 0.097 *agsiso+ (explicit uncharged)* - 0.081 *achdda- (implicit charged)* - 0.054 *msagha (implicit uncharged)* + 0.046 *gsalpi- (explicit charged)* - 0.061 *stwist- (explicit charged)* - 0.063 *msgsag (implicit charged)* (S18) | | | | | | | |

n = 42
