# Supplementary material for: Development of Ensemble Steric and Electrostatic Chirality (ESEC) descriptors for modelling chromatographic enantioseparations
Source: PLoS One. 2025 Oct 17;20(10):e0333635. doi: 10.1371/journal.pone.0333635 (PMC12533851; doi:10.1371/journal.pone.0333635)
Supplement: S7 Table — (DOCX) [file pone.0333635.s021.docx]

**S7 Table.** **sMLR models for *α_RS_* and log *α_RS_* using only the windowed descriptors acquired from explicit solvent MD simulations.**

| Chiral descriptors calculated from MD simulations in **explicit** solvent  **windowed charged and uncharged descriptors** | | | | | | | | | |
| --- | --- | --- | --- | --- | --- | --- | --- | --- | --- |
| **Solvent system** | **Descriptors** | **RMSECV_N_** | **RMSEC_N_** | **r^2^** | **q^2^** | **Prediction error (%)** | **Accurate predictions** | **Correct predictions** | **Elution sequence** |
| Water  /ACN | 5 | 0.113 | 0.0899 | 0.7199 | 0.4531 | 11.47 | 8/42 | 19/42 | 16/23 |
|  | **Equation** | Log *α_RS_* = -0.020 - 0,080 *msgshb- (uncharged)* + 0,073 *agsiso+ (uncharged)* – 0,029 *achdhb- (charged)* – 0,040 *msagpi- (uncharged)* - 0,028 *msgspi- (uncharged)* (S19) | | | | | | | |
| Water  /ACN | 8 | 0.0889 | 0.0683 | 0.8305 | 0.7132 | 7.45 | 17/42 | 25/42 | 21/23 |
|  | **Equation** | *α_RS_* = -0.98 - 0.12 *msgshb- (uncharged)* + 0.20 *agsiso+ (uncharged)* - 0.10 *achdhb- (charged)* - 0.074 *msgsha+ (charged)* - 0.060 *acsiso- (charged)* + 0.13 *agsiso- (uncharged)* + 0.047 *chpida+ (uncharged)* – 0.087 *gspida+ (uncharged)* (S20) | | | | | | | |
